# Immunogenicity and therapeutic targeting of a public neoantigen derived from mutated *PIK3CA*

**DOI:** 10.1038/s41591-022-01786-3

**Published:** 2022-04-28

**Authors:** Smita S. Chandran, Jiaqi Ma, Martin G. Klatt, Friederike Dündar, Chaitanya Bandlamudi, Pedram Razavi, Hannah Y. Wen, Britta Weigelt, Paul Zumbo, Si Ning Fu, Lauren B. Banks, Fei Yi, Enric Vercher, Inaki Etxeberria, Watchain D. Bestman, Arnaud Da Cruz Paula, Ilinca S. Aricescu, Alexander Drilon, Doron Betel, David A. Scheinberg, Brian M. Baker, Christopher A. Klebanoff

**Affiliations:** 1 Human Oncology and Pathogenesis Program (HOPP), Immuno-Oncology Service, Memorial Sloan Kettering Cancer Center, New York, NY USA; 2 Center for Cell Engineering, Memorial Sloan Kettering Cancer Center, New York, NY USA; 3 Parker Institute for Cancer Immunotherapy, New York, NY USA; 4 Department of Chemistry and Biochemistry, University of Notre Dame, South Bend, IN USA; 5 Harper Cancer Research Institute, University of Notre Dame, South Bend, IN USA; 6 Molecular Pharmacology Program, Sloan Kettering Institute, Memorial Sloan Kettering Cancer Center, New York, NY USA; 7 Department of Physiology and Biophysics, Weill Cornell Medicine, New York, NY USA; 8 Applied Bioinformatics Core, Weill Cornell Medicine, New York, NY USA; 9 Marie-Josée and Henry R. Kravis Center for Molecular Oncology, Memorial Sloan Kettering Cancer Center, New York, NY USA; 10 Department of Medicine, Memorial Sloan Kettering Cancer Center, New York, NY USA; 11 Department of Medicine, Division of Hematology and Medical Oncology, Weill Cornell Medicine, New York, NY USA; 12 Department of Pathology, Memorial Sloan Kettering Cancer Center, New York, NY USA; 13 Department of Surgery, Memorial Sloan Kettering Cancer Center, New York, NY USA; 14 Early Drug Development Service, Memorial Sloan Kettering Cancer Center, New York, NY USA; 15 Institute for Computational Biomedicine, Weill Cornell Medicine, New York, NY USA; 16 Cell Therapy Service, Memorial Sloan Kettering Cancer Center, New York, NY USA

**Keywords:** Sequencing, Tumour immunology, Tumour immunology, Cancer immunotherapy, T cells

## Abstract

Public neoantigens (NeoAgs) represent an elite class of shared cancer-specific epitopes derived from recurrently mutated driver genes. Here we describe a high-throughput platform combining single-cell transcriptomic and T cell receptor (TCR) sequencing to establish whether mutant *PIK3CA*, among the most frequently genomically altered driver oncogenes, generates an immunogenic public NeoAg. Using this strategy, we developed a panel of TCRs that recognize an endogenously processed neopeptide encompassing a common *PIK3CA* hotspot mutation restricted by the prevalent human leukocyte antigen (HLA)-A*03:01 allele. Mechanistically, immunogenicity to this public NeoAg arises from enhanced neopeptide/HLA complex stability caused by a preferred HLA anchor substitution. Structural studies indicated that the HLA-bound neopeptide presents a comparatively ‘featureless’ surface dominated by the peptide’s backbone. To bind this epitope with high specificity and affinity, we discovered that a lead TCR clinical candidate engages the neopeptide through an extended interface facilitated by an unusually long CDR3β loop. In patients with diverse malignancies, we observed NeoAg clonal conservation and spontaneous immunogenicity to the neoepitope. Finally, adoptive transfer of TCR-engineered T cells led to tumor regression in vivo in mice bearing *PIK3CA*-mutant tumors but not wild-type *PIK3CA* tumors. Together, these findings establish the immunogenicity and therapeutic potential of a mutant *PIK3CA*-derived public NeoAg.

## Abstract (web summary)

A new high-throughput platform to find rare T cells that can recognize shared cancer neoantigens identifies T cell receptors specific for a conserved, immunogenic and therapeutically actionable epitope in mutant PI3Kα, one of the most common driver oncogenes

## Main

Neoantigens (NeoAgs)—HLA-bound peptides resulting from non-synonymous somatic mutations (NSSMs)—are a major class of cancer regression antigens^1^. Most NeoAgs result from random passenger mutations^2^. Patient-specific, or ‘private’, NeoAgs pose a significant challenge to therapeutically target because they require customization^3^. Furthermore, private NeoAgs are subject to clonal heterogeneity, a major mechanism of immunotherapy resistance^4–6^. In contrast, NeoAgs resulting from gain-of-function mutations in driver genes would generally be clonally conserved because the source proteins directly contribute to cancer cell fitness^7^. If a NeoAg resulting from a driver hotspot mutation is restricted by a prevalent HLA allele, this elite subset of cancer-specific epitopes would be shared among patients with cancer, creating a ‘public’ NeoAg^8^.

Only a limited number of public NeoAgs have been identified to date^9–16^. Critically, none has been reported arising from mutant (Mut) *PIK3CA* (the gene encoding phosphoinositide 3-kinase alpha, PI3Kα), which is among the most common genetically altered driver oncogenes^17–19^. Small-molecule PI3K inhibitors cause cancer regression, validating PI3Kα as a therapeutic target^20^. However, these inhibitors are not curative and can be associated with significant on-target/off-tumor toxicities. Novel immunotherapies that target Mut PI3Kα while sparing healthy tissues could have broad utility for many patients with cancer. Here we report on a high-throughput platform combining single-cell transcriptomic and TCR sequencing to test whether Mut *PIK3CA* generates an immunogenic and therapeutically actionable public NeoAg.

## Results

### Public NeoAg TCR discovery platform

**Fig. 1:**
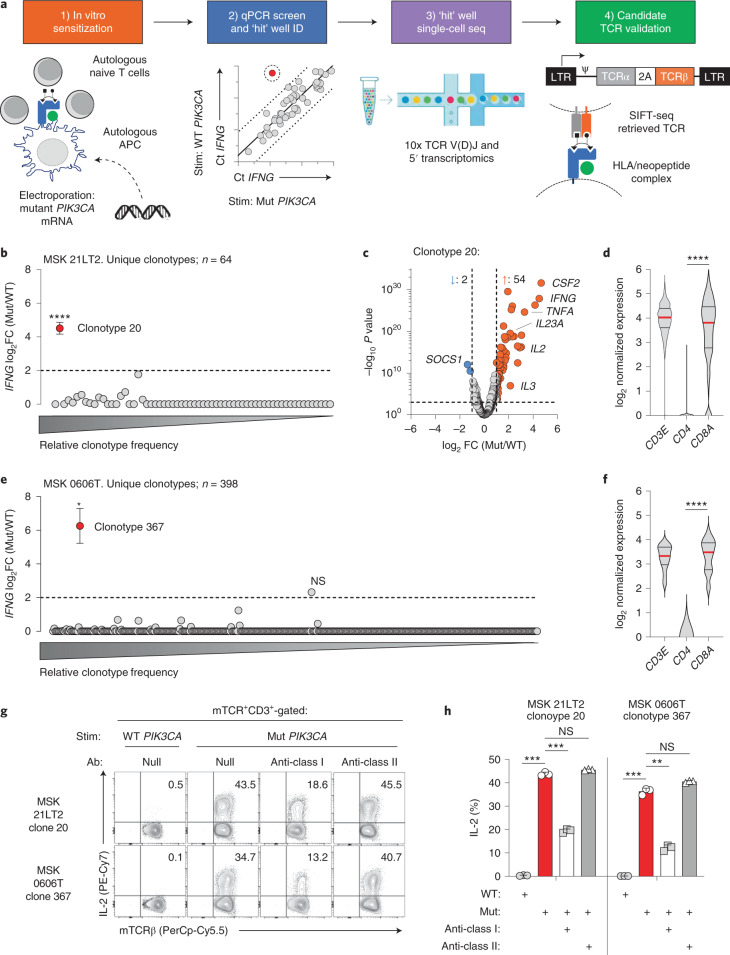
Development and validation of the SIFT-seq discovery platform for Mut *PIK3CA*-specific TCRs. **a**, Schematic overview of the SIFT-seq TCR discovery platform. **b**, log_2_ fold change (FC) ratio of *IFNG* transcripts from *n* = 64 unique TCR clonotypes identified using single-cell RNA and V(D)J TCR sequencing from screen-positive ‘hit’ well MSK 21LT2. Matched aliquots of sensitized T cells from HD1 were stimulated with *PIK3CA* (H1047L) (Mut) or WT *PIK3CA* before single-cell sequencing. The mean *IFNG* ratio for all evaluable TCR clonotypes is shown. Statistical analyses were performed for all clonotypes with a minimal ratio of ≥2 (dashed line). The *x* axis indicates the relative frequencies of individual TCR clonotypes. ****P = 1.37 × 10^−28^ using two-sided Welch’s *t*-test. **c**, Volcano plot displaying global transcriptomic changes for MSK 21LT2 clonotype 20 after stimulation with Mut versus WT *PIK3CA*. Vertical and horizontal dashed lines indicate thresholds for log_2_ gene expression FC and statistical significance, respectively. Orange and blue dots represent significantly upregulated and downregulated genes after Mut *PIK3CA* stimulation, respectively. **d**, Violin plots depicting transcript levels for the lineage markers *CD3E*, *CD4* and *CD8A* from MSK 21LT2 clonotype 20. ****P < 0.0001 using two-sided Student’s *t*-test. **e**, log_2_ FC ratio of *IFNG* transcripts from *n* = 398 unique TCR clonotypes identified using single-cell sequencing from screen-positive ‘hit’ well MSK 06006T derived from HD2. *P = 1.41 × 10^−5^ using two-sided Welch’s *t*-test. NS, not significant. **f**, Violin plots depicting lineage marker transcript expression for MSK 0606T clonotype 367. ****P < 0.0001 using two-sided Student’s *t*-test. Representative FACS plots (**g**) and summary bar graph (**h**) (*n* = 3 biologically independent replicates per condition) displaying the frequency of intracellular IL-2 production in polyclonal T cells following retroviral transduction with SIFT-seq-retrieved TCR candidates. The reconstructed TCR expresses a murine constant chain (mTCR), enabling detection with an anti-mTCR-specific antibody. Transduced T cells (live^+^mTCR^+^CD3^+^) were co-cultured with autologous moDCs electroporated with mRNA encoding Mut or WT *PIK3CA* in the absence or presence of pan-HLA class I or class II blocking antibodies. ***P < 0.001, **P = 0.0036 and NS using two-sided Student’s *t*-test with Bonferroni correction. **b**,**e**,**h**, Symbols and bar graphs are displayed as mean ± s.e.m. **d**,**f**, Violin distributions are centered around the median (red horizontal line) with quartile ranges displayed above and below (dashed horizontal lines). The maxima and minima are represented by the top and bottom of each plot.

We developed a high-throughput method to test whether Mut PI3Kα is immunogenic and to retrieve TCR gene sequences that confer specificity to this NeoAg (Fig. 1a). The platform proceeds in four steps. First, healthy donor (HD) naive T cells (T_N_) undergo in vitro sensitization (IVS) using autologous monocyte-derived dendritic cells (moDCs) electroporated with mRNA encoding Mut *PIK3CA*. Second, matched aliquots from sensitized wells are re-stimulated with moDCs expressing Mut or wild-type (WT) *PIK3CA* to identify ‘hit’ wells containing T cells with preferential activation to the Mut antigen using a qPCR screen. Third, T cells from ‘hit’ wells are again re-stimulated to identify reactive clonotypes and retrieve their TCR gene sequences. This step, which we have termed stimulation-induced functional TCR sequencing (SIFT-seq), uses combined single-cell TCR V(D)J and RNA sequencing. Finally, TCR clonotypes that express activation-associated transcripts in response to Mut but not WT *PIK3CA* are reconstructed for functional validation.

Most *PIK3CA* mutations occur at three hotspots, with H1047 being the most common^21^. Healthy donor-derived T_N_ underwent IVS by moDCs transfected with genes encoding the four most frequent *PIK3CA* hotspot mutations (H1047L/R, E542K and E545K). The qPCR screen identified a single well (21LT2) that preferentially upregulated *IFNG* in response to *PIK3CA* (H1047L) (henceforth Mut *PIK3CA*), which was subsequently subjected to SIFT-seq. To identify candidate TCR sequences, we plotted the ratio of *IFNG* produced under Mut and WT stimulation conditions for each clonotype (Fig. 1b). Among *n* = 64 clonotypes, only clonotype 20 significantly upregulated *IFNG* to Mut *PIK3CA*. Global transcriptomic analysis of clonotype 20 revealed additional Mut-specific gene expression changes (Fig. 1c), whereas all other clonotypes retained a quiescent profile (Supplementary Fig. 1a). Lineage marker assessment revealed expression of *CD3E* and *CD8A* but minimal to no *CD4* (Fig. 1d), suggesting that clonotype 20 was human leukocyte antigen (HLA) class I (HLA-I) restricted. We performed an additional run using peripheral blood mononuclear cells (PBMCs) from a second HD (HD2). A single qPCR screen-positive well (0606T) was identified and subjected to SIFT-seq. Among *n* = 398 clonotypes, only clonotype 367 selectively upregulated *IFNG* (Fig. 1e) and other TCR activation-induced genes (Supplementary Fig. 1b) in response to Mut *PIK3CA*. Similar to clonotype 20 from HD1, clonotype 367 expressed *CD8A* but minimal *CD4* (Fig. 1f), suggesting that this TCR was also HLA-I restricted.

Polyclonal T cells retrovirally (RV) transduced with SIFT-seq-retrieved TCR sequences were co-cultured with autologous moDCs transfected with Mut or WT *PIK3CA*. Expression of TCRs from 21LT2 clonotype 20 and 0606T clonotype 367 resulted in polyfunctional cytokine production exclusively in response to Mut *PIK3CA* (Fig. 1g and Supplementary Fig. 2), validating the SIFT-seq approach. As controls, we synthesized TCRs from the dominant clonotypes in each screen-positive well in addition to clonotypes that expressed *IFNG* under both Mut and WT stimulation conditions and confirmed that these do not confer Mut *PIK3CA* reactivity (Supplementary Figs. 1, 3 and 4). To determine whether the Mut *PIK3CA*-specific TCRs were HLA-I or HLA-II restricted, we tested their function in the presence of HLA class-specific blocking antibodies. Cytokine release by both TCRs was attenuated exclusively by an HLA-I blocking antibody (Fig. 1g,h), demonstrating that SIFT-seq correctly predicted the HLA class restriction of Mut-specific clonotypes. We conclude that the SIFT-seq platform can reproducibly identify TCR sequences that confer specificity to a *PIK3CA* hotspot mutation.

### HLA restriction and functionality of *PIK3CA* public NeoAg TCRs

**Fig. 2:**
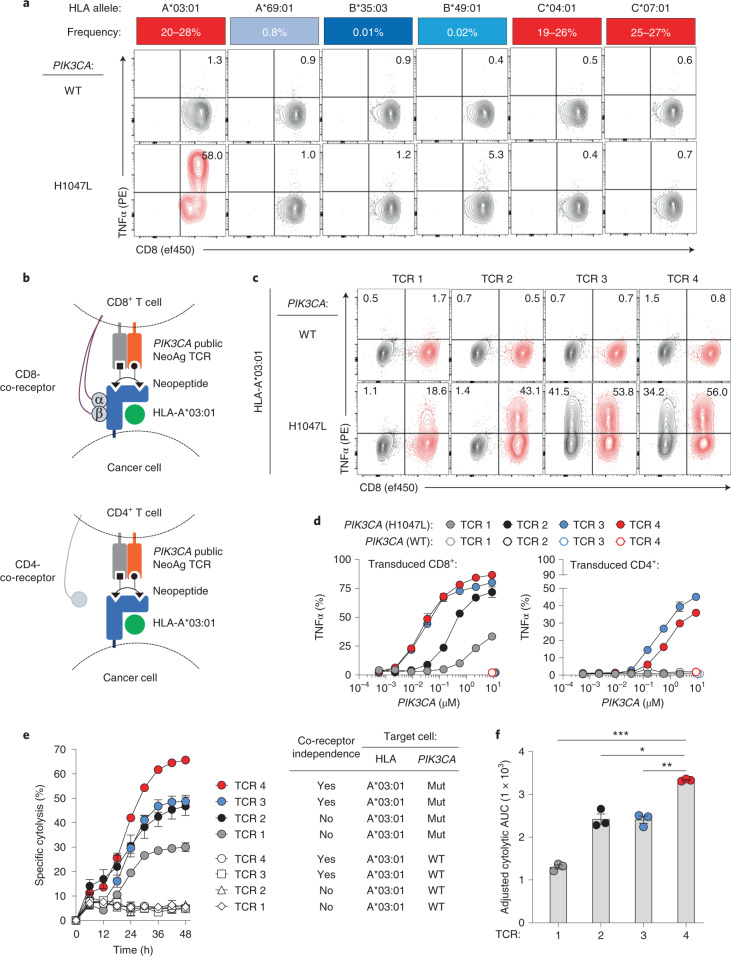
HLA restriction and functional characterization of *PIK3CA* public NeoAg-specific TCRs. **a**, Deconvolution of the HLA-I restriction element for the MSK 21LT2 clonotype 20 TCR. The frequency of individual HLA-I alleles expressed by HD1 in North American and European populations is displayed as a heat map. FACS plots show the frequency of CD8^+^ TCR-transduced T cells that secrete TNFα after co-culture with HLA-I mono-allelic cell lines expressing WT or Mut *PIK3CA*. **b**, Cartoon illustrating the experimental design to assess the co-receptor dependence and functionality of SIFT-seq-retrieved *PIK3CA* public NeoAg TCR panel members. TCRs 1–4 were individually RV transduced into enriched CD8^+^ or CD4^+^ T cells and co-cultured with target cells co-expressing HLA-A*03:01 and either WT or Mut *PIK3CA*. **c**, Representative FACS plots of CD4^+^ (black) and CD8^+^ (red) T cells expressing individual PIK3CA public NeoAg TCR panel members after co-culture with HLA-A*03:01^+^ target cells that express either WT or Mut *PIK3CA*. Numbers within each plot indicate the frequency of TNFα-producing TCR-transduced CD4^+^ (upper left quadrant) or CD8^+^ (upper right quadrant) T cells. The FACS plots shown in **a** and **c** are pre-gated on live^+^mTCR^+^ T cells. **d**, The functional avidity of CD8^+^ (left) or CD4^+^ (right) T cells individually transduced with TCRs 1–4. Transduced T cells were co-cultured with an HLA-I mono-allelic cell line expressing HLA-A*03:01 and electroporated with indicated concentrations of WT or Mut *PIK3CA* mRNA. **e**, Kinetic impedance-based lytic assay measuring the % specific cytolysis of HLA-A*03:01^+^ target cells expressing WT or Mut *PIK3CA*. **f**, Adjusted cytolytic AUC values indicating the cumulative cytolytic capacity of TCRs 1–4 against A*03:01^+^/Mut *PIK3CA*^+^ target cells. ***P = 0.001, **P = 0.01 and *P = 0.02; two-sided Student’s *t*-test was used for statistical analysis. Data shown in **d**, **e** and **f** are representative of two independent experiments using *n* = 3 biologically independent replicates per condition per independent experiment. Symbols and bar graphs are displayed as mean ± s.e.m. AUC, area under the curve.

**Extended Data Fig. 1:**
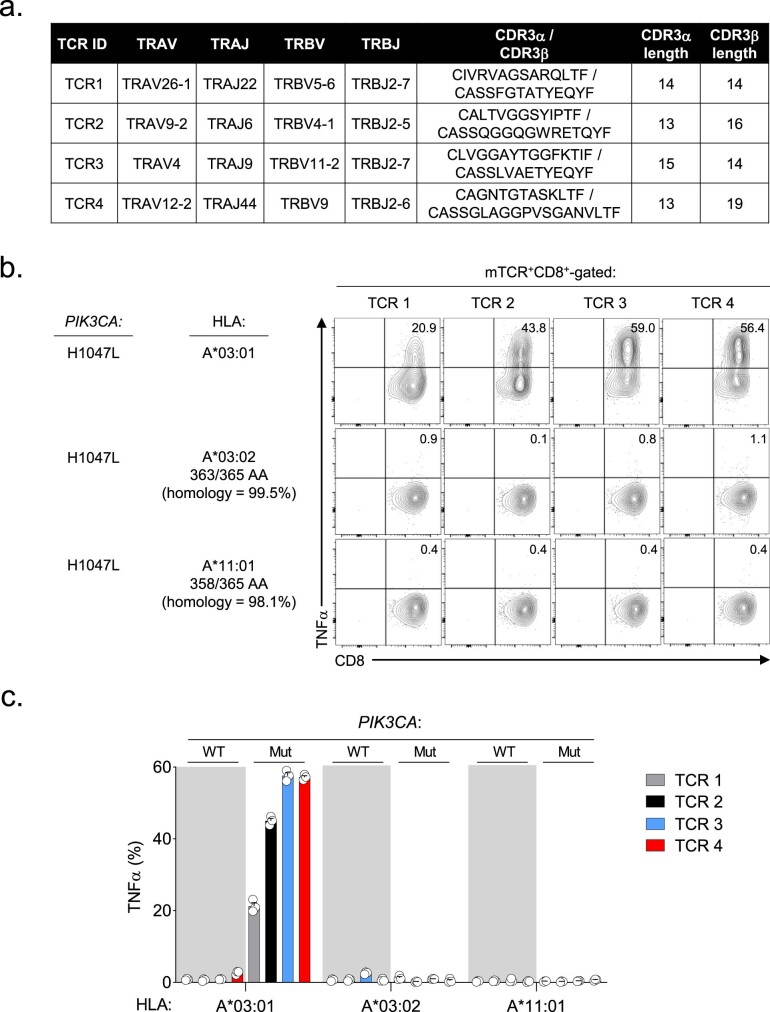
Variable chain gene sequences and HLA specificity of a *PIK3CA* public NeoAg-specific TCR panel. (**a**) Table listing *TRAV*, *TRAJ*, *TRBV* and *TRBJ* gene segment composition, CDR3 sequences, and CDR3 lengths for a panel of *PIK3CA* public NeoAg TCRs retrieved using the SIFT-seq discovery platform. (**b**) Representative FACS plots and (**c**) summary bar graphs demonstrating the specificity of *PIK3CA* public NeoAg TCRs 1-4 for HLA-A03 supertype members HLA-A*03:01, HLA-A*03:02, and HLA-A*11:01. The number of shared amino acids and % homology of each supertype member to HLA-A*03:01 are shown. T cells transduced with individual TCR panel members were co-cultured with mono-allelic cell lines co-expressing the indicated HLA-A03 supertype member and either Mut or WT *PIK3CA*. Numbers within each FACS plot and bar graph indicate the frequency of TNFα producing T cells after pre-gating on live^+^mTCR^+^CD8^+^ cells. Bar graphs displayed as mean ± SEM using *n* = 3 biologic replicates per condition. Data shown is representative of 2 independent experiments.

To resolve which HLA-I allele is required for 21LT2 TCR clonotype 20 recognition, we generated mono-allelic cells that display individual HLA-I molecules from HD1 together with Mut or WT *PIK3CA* (Fig. 2a). Co-culture of these targets with T cells transduced with 21LT2 TCR clonotype 20 revealed cytokine production exclusively in the context of Mut *PIK3CA* and HLA-A*03:01. This HLA-I allele is among the most common in North America and Europe, occurring in 20–28% of individuals^22^. Using a similar approach, we resolved that 0606T TCR clonotype 367 is also restricted by HLA-A*03:01. Based on these data, we performed SIFT-seq on two additional HLA-A*03:01^+^ HDs (Supplementary Table 1) and retrieved two additional TCRs that recognize the same Mut *PIK3CA*/HLA-I combination. TCRs reactive against alternative *PIK3CA* mutations included in each screen were not detected. The resulting Mut *PIK3CA* TCR panel (TCRs 1–4) uses distinct variable segments, unique complementarity-determining region 3 (CDR3) sequences and diverse CDR3 lengths (Extended Data Fig. 1a).

Polyclonal CD8^+^ and CD4^+^ T cells were transduced with individual TCR panel members to characterize their specificity, functional avidity and cytolytic capacity (Fig. 2b). In the context of HLA-A*03:01, all members exhibited absolute specificity for Mut *PIK3CA* (Fig. 2c). HLA-A*03:01 is a member of the HLA-A3 supertype, a group of homologous HLA-I molecules characterized by overlapping peptide-binding repertoires^23^. Within this supertype, HLA-A*03:01 shares the highest homology with HLA-A*03:02 (99.5%) and HLA-A*11:01 (98.1%). To determine if these related HLA-I alleles trigger Mut-specific function, we co-cultured target cells expressing HLA-A*03:01, HLA-A*03:02 or HLA-A*11:01 with T cells transduced with TCRs 1–4. All TCR panel members solely recognized Mut *PIK3CA* in the context of HLA-A*03:01, highlighting each receptor’s selectivity for a single HLA-I allele (Extended Data Fig. 1b,c).

We next compared each TCR’s functionality when transduced into CD8^+^ or CD4^+^ T cells. When expressed in CD8^+^ T cells, TCRs 3 and 4 triggered significantly greater Mut-specific cytokine production compared to TCRs 1 and 2 (Fig. 2c and Supplementary Fig. 5a). When transduced into CD4^+^ T cells, only TCRs 3 and 4 triggered T cell function, indicating co-receptor independence (Fig. 2c). The capacity of a TCR to function in a co-receptor-independent manner correlates with a receptor’s intrinsic affinity to the peptide/HLA complex^24^ and clinical anti-tumor efficacy^25–28^. Next, we quantified each TCR’s relative antigen sensitivity by determining their functional avidity. In CD8^+^ T cells, TCRs 3 and 4 had lower EC_50_ values to Mut *PIK3CA* compared to TCRs 1 and 2 (Fig. 2d and Supplementary Fig. 5b); in CD4^+^ T cells, only TCRs 3 and 4 were functional.

Finally, we measured the capacity of CD8^+^ T cells transduced with TCRs 1–4 to mediate Mut-specific cell killing. All TCRs mediated cytolysis of Mut *PIK3CA*^+^/HLA-A*03:01^+^ target cells but spared HLA-A*03:01^+^ cells expressing WT *PIK3CA* (Fig. 2e). In repeated experiments, TCR4 exhibited superior lytic efficiency relative to other panel members (Fig. 2f). Together, these data establish the existence of an immunogenic *PIK3CA* public NeoAg that is targetable using TCRs with diverse functional attributes.

### Mechanism of immunogenicity for a *PIK3CA* public NeoAg

**Fig. 3:**
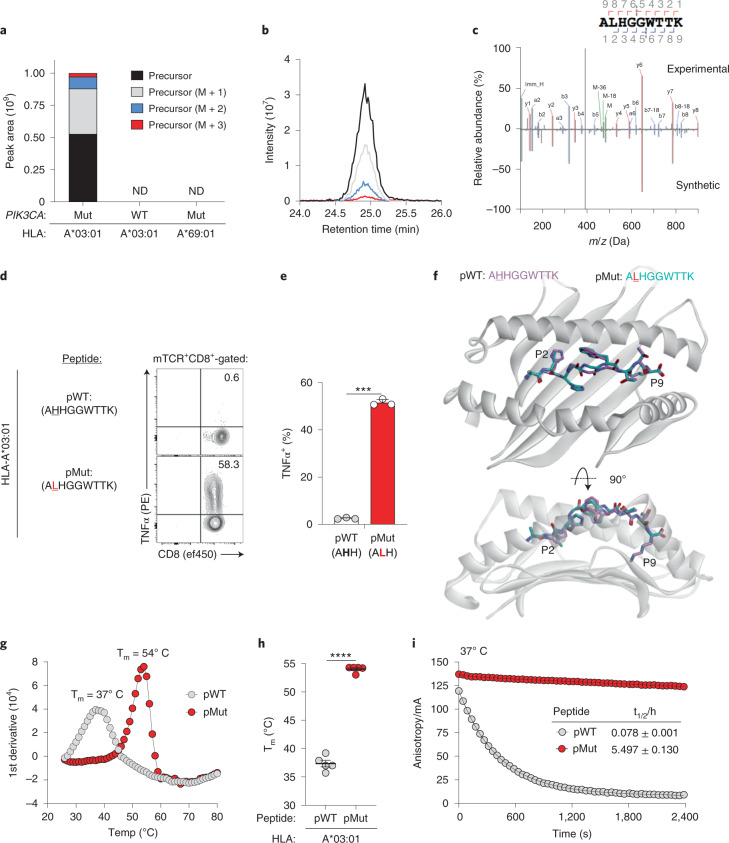
Mechanism of immunogenicity for a *PIK3CA* public NeoAg. **a**, Skyline analysis of HLA-I-bound peptides resulting from an LC–MS/MS-based immune-peptidomic screen. Relative abundance of precursor ions derived from peptides encompassing the PI3Kα protein’s 1047 position eluted from the indicated HLA-A mono-allelic cell lines. Cells expressed either WT *PIK3CA* or Mut *PIK3CA* (H1047L). ND indicates conditions in which no PI3Kα-derived AA sequences encompassing the 1047 hotspot position were experimentally detected. **b**, Corresponding chromatographic retention times of precursor ions derived from a Mut PI3Kα peptide eluted from HLA-A*03:01^+^/Mut *PIK3CA*^+^ cells. **c**, Mirror plot displaying the MS2 spectra of the Mut PI3Kα-derived public neoepitope (ALHGGWTTK; pMut; top) eluted from HLA-A*03:01^+^/Mut *PIK3CA*^+^ cells and a synthetically generated peptide (bottom). Peaks represent *b* ions in blue and *y* ions in red. Representative intracellular FACS analysis (**d**) and summary bar graph (**e**) for TNFα production in T cells transduced with TCR4 and co-cultured with HLA-A*03:01^+^ target cells pulsed with 1 μM of pMut versus pWT. Results shown after gating on live^+^mTCR^+^CD8^+^ lymphocytes. Bar graph is displayed as mean ± s.e.m. using *n* = 3 biologically independent replicates per condition. ***P = 0.001 using two-sided Student’s *t*-test. **f**, Structural superimposition of the pMut and pWT peptides bound to HLA-A*03:01. The conformations of pMut and pWT peptides are nearly identical with all α carbon atoms superimposing with a root mean square deviation of 0.73 Å. Representative thermal melt curves (**g**) and summary scatter plot (**h**) displaying the melting temperatures (T_m_) of the pMut and pWT/HLA-A*03:01 complexes using differential scanning fluorimetry. Symbols are displayed as mean ± s.e.m. using *n* = 5 independently performed experiments. ****P < 0.0001 using two-sided Student’s *t*-test. **i**, Dissociation of a fluorescently labeled pMut or pWT from soluble HLA-A*03:01 complexes at 37 °C using fluorescence anisotropy. Solid lines show fits to exponential decay functions. Half-lives (t_1/2_) are shown for each peptide ± s.e.m. Data are representative of *n* = 3 technical replicates per condition per time point. mA, millianisotropy.

**Extended Data Fig. 2:**
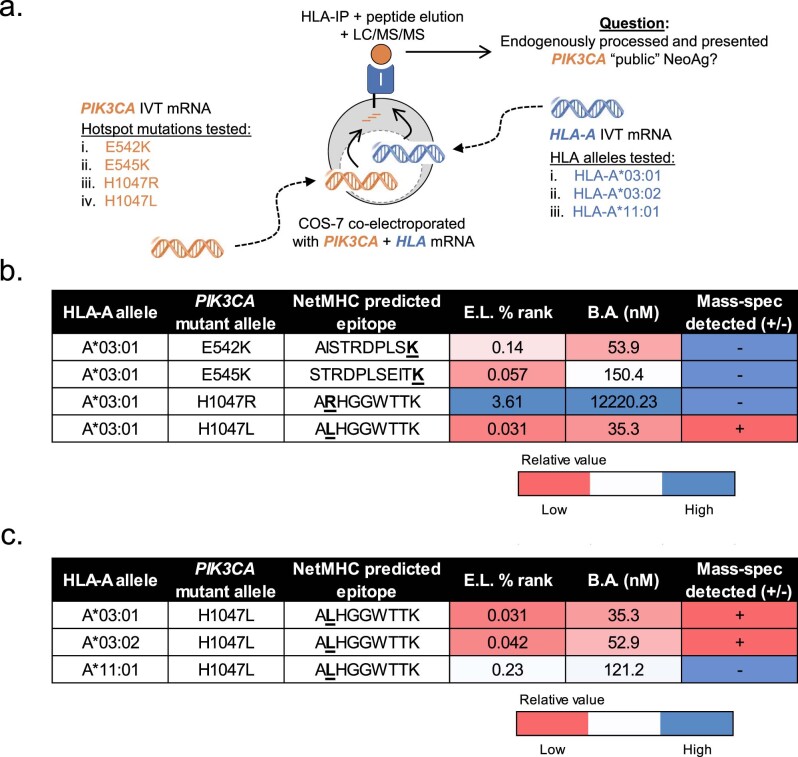
An immuno-peptidomic screen for HLA-A3 supertype-restricted public neoantigens resulting from common *PIK3CA* hotspot mutations. (**a**) Schematic overview of an HLA immunoprecipitation/liquid chromatography tandem mass-spectrometry (HLA-IP/MS) screen to identify endogenously processed and presented peptides resulting from the *PIK3CA* hotspot mutations E542K, E545K, H1047R and H1047L in the context of various HLA-A3 supertype members. COS-7 was co-electroporated with IVT mRNA encoding either HLA-A*03:01, HLA-A*03:02, or HLA-A*11:01 and an individual *PIK3CA* hotspot mutation. (**b**) Summary table listing the NetMHCpan 4.1 predicted peptide sequence, eluted ligand % rank, and binding affinity (B.A.) of various peptides containing a PI3Kα hotspot mutations to HLA-A*03:01. Whether the peptide was detected by HLA-IP/MS is listed in the adjacent column. (**c**) Summary table listing the NetMHCpan 4.1 predicted peptide sequence, eluted ligand (E.L.) % rank, and binding affinity (B.A.) of the ALHGGWTTK peptide (pMut) to HLA-A*03:01, HLA-A*03:02, and HLA-A*11:01. Whether the peptide was detected by HLA-IP/MS is listed in the adjacent column. Heat map colors in (**b**) and (**c**) indicate the relative value for the NetMHCpan 4.1 prediction (red = low, blue = high) or whether the peptide sequence was detected by HLA-IP/MS (red = yes, blue = no).

We next determined the sequences of endogenously processed PI3Kα peptides presented by HLA-A*03:01. We performed HLA-immune precipitation liquid chromatography–tandem mass spectrometry (HLA-IP LC–MS/MS) on cells that express individual HLA-I alleles and either WT *PIK3CA* or one of the four *PIK3CA* mutations we tested by SIFT-seq. Skyline and chromatogram analyses revealed PI3Kα-derived amino acid (AA) sequences encompassing a hotspot Mut position exclusively from cells that co-express HLA-A*03:01 and *PIK3CA* (H1047L) (Fig. 3a,b and Extended Data Fig. 2). No peptides were detected from cells that express HLA-A*03:01 and WT *PIK3CA* or *PIK3CA* (H1047L) and a mismatched HLA-A allele. Although NetMHCpan 4.1 predicts two high-affinity HLA-A*03:01-restricted peptides surrounding PI3Kα E542K and E545K^29^, neither epitope was detected (Extended Data Fig. 2b). Similarly, although NetMHCpan 4.1 also predicts 15 high-affinity WT PI3Kα-derived peptides that bind HLA-A*03:01, we detected only one (Supplementary Fig. 6). To exclude that a high-affinity Mut peptide limited the detection of weaker-binding WT peptides, we repeated this experiment after subdividing WT *PIK3CA* into overlapping minigenes and uncovered only two additional WT *PIK3CA* peptides (Supplementary Fig. 6). To establish whether the specificity of our TCR panel members for HLA-A*03:01 was attributable to differential antigen presentation by closely related HLA-A3 superfamily members, we performed HLA-IP LC–MS/MS on cells that co-express *PIK3CA* (H1047L) and either HLA-A*03:02 or HLA-A*11:01. We discovered that HLA-A*03:02, but not HLA-A*11:01, was capable of presenting a Mut peptide (Extended Data Fig. 2c).

Deconvolution of the MS/MS spectrum from HLA-A*03:01^+^/*PIK3CA* (H1047L)^+^ cells divulged a single Mut-containing peptide with the His→Leu substitution occurring at the second position (P2) (ALHGGWTTK; pMut). The sequence of the eluted neopeptide was validated by matching its MS/MS spectra with that of a synthetic peptide (Fig. 3c). Co-culture of Mut *PIK3CA*-specific T cells with HLA-A*03:01^+^ target cells pulsed with pMut confirmed that this sequence, but not its WT counterpart (AHHGGWTTK; pWT), triggered T cell function (Fig. 3d,e).

We next sought to establish the physical basis for *PIK3CA* public NeoAg immunogenicity. We crystallized and determined the X-ray structures of the pMut and pWT peptides bound to HLA-A*03:01 at resolutions of 2.0 Å and 2.1 Å, respectively (Fig. 3f, Supplementary Fig. 7 and Supplementary Table 2). The pMut and pWT adopt nearly identical conformations when bound by HLA-A*03:01 (root mean square deviation = 0.72 Å). In both structures, the P6 Trp packs between the peptide backbone and the HLA-I α1 helix, whereas the P7 Thr packs against the α2 helix. Together with the P4/P5 Gly residues, this results in a largely ‘featureless’ central peptide region dominated by the peptide’s backbone. The overall similarities of the two complexes suggest that structural differences alone likely cannot explain the immunogenic potential of the *PIK3CA* public NeoAg.

Because the *PIK3CA* public NeoAg introduces a preferred P2 anchor residue for HLA-A*03:01 (ref. ^30^), we next quantified the stability of the pMut and pWT peptides bound to HLA-A*03:01 using differential scanning fluorimetry. Consistent with the introduction of an optimal P2 anchor, we discovered that the melting temperature (T_m_) of the pMut/HLA-I complex was significantly higher than the pWT/HLA-I complex (*P* < 0.0001; Fig. 3g,h). Similar results were obtained with HLA-A*03:02 (Supplementary Fig. 8), suggesting that the specificity for the TCR panel members for HLA-A*03:01 over HLA-A*03:02 might result from engagement of a key residue in HLA-A*03:01 and/or subtle conformational differences in the HLA-bound pMut.

Improvements in peptide-binding affinity are often associated with longer p/HLA half-lives (t_1/2_). Therefore, we next resolved the kinetic stability of the two p/HLA-A*03:01 complexes at physiologic temperature (37 °C) using fluorescence anisotropy. At 37 °C, the pMut/HLA-I complex had a significantly longer t_1/2_ compared to pWT (t_1/2_ = 5.497 hours ± 0.130 versus 0.078 hours ± 0.001; Fig. 3I). We conclude that the formation of the *PIK3CA* public NeoAg is driven by the creation of a peptide sequence containing a preferred HLA-I anchor residue, resulting in a stable, high-affinity p/HLA-I complex with a prolonged t_1/2_.

### Structural correlates of *PIK3CA* public NeoAg TCR specificity

**Fig. 4:**
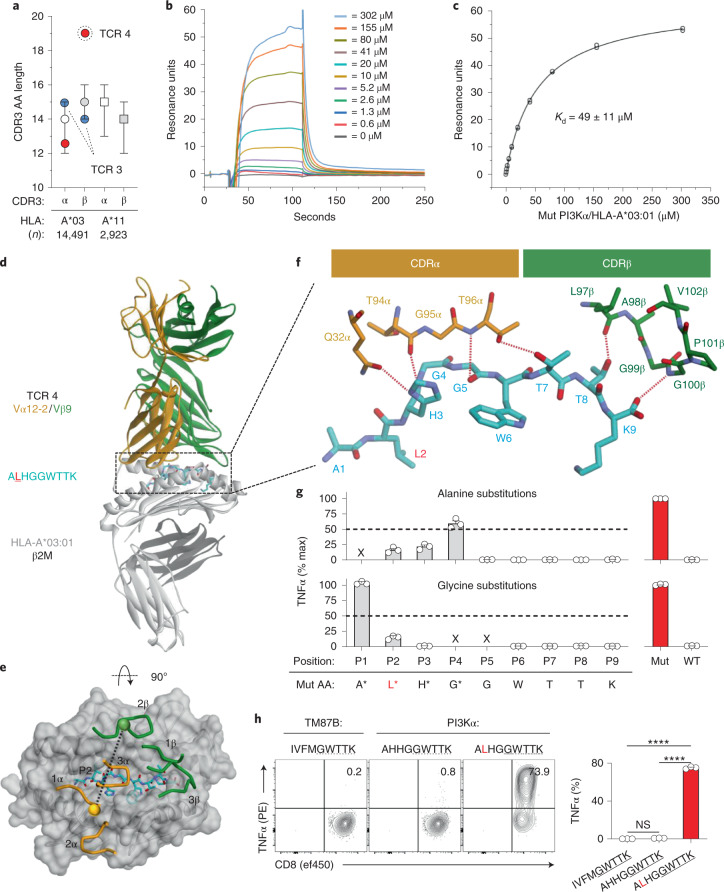
Structural correlates of affinity and specificity for a *PIK3CA* public NeoAg-specific TCR. **a**, CDR3α and CDR3β AA lengths of *PIK3CA* public NeoAg-specific TCR3, TCR4 and a panel of *n* = 17,414 HLA-A*03:01-restricted and HLA-A*11:01-restricted TCR sequences. Results shown as median ± interquartile range. Representative SPR sensorgram (**b**) and steady-state binding equilibrium (**c**) measuring the dissociation constant (*K*_d_) for TCR4 to the pMut/HLA-I complex. Results shown are the average ± s.d. of *n* = 4 independent experiments. **d**, Structural overview of the TCR4 pMut/HLA-A*03:01 ternary complex at 3.1-Å resolution. The color scheme is indicated and replicated throughout. **e**, Top view of the pMut/HLA-A*03:01 complex displaying the crossing angle and positions of the six CDR loops of TCR4. Spheres represent the centers of mass of the TCR’s Vα and Vβ domains. **f**, AAs of TCR4’s CDRα and CDRβ loops that interact with the pMut peptide. Hydrogen bonds (*n* = 6) are indicated by red dashed lines. AAs are identified by standard one-letter codes followed by position number. The side chains of contacting residues from the TCR are also identified by AA and hemi-chain position number. **g**, Identification of TCR4’s peptide recognition motif using alanine and glycine scanning. Intracellular FACS for TNFα production to Ala (upper) or Gly (lower) substituted peptides. TCR4-transduced T cells (identified by gating on mTCR^+^ lymphocytes) were co-cultured with HLA-A*03:01^+^ targets pulsed with 1 μM of indicated peptides. Results are shown as the mean ± s.e.m. percent maximum response relative to the native pMut peptide using *n* = 3 biologic replicates per condition. ‘x’ indicates positions not amenable to substitution. **h**, Measurement of the cross-reactivity potential of TCR4. TCR4-transduced T cells were co-cultured with HLA-A*03:01^+^ targets pulsed with 1 μM of peptides containing the motif ‘x-x-x-x-G-W-T-T-K’. Results are shown as mean ± s.e.m. using *n* = 3 biologic replicates per condition. ****P < 0.0001 using two-sided Student’s *t*-test with Bonferroni correction. NS, not significant.

TCR specificity is determined by six CDR loops, especially the hypervariable CDR3 loops^31,32^. We noted that TCR4 has a significantly longer (19 AAs) CDR3β loop than TCRs 1–3 (range, 14–16 AAs) and a panel of more than 17,000 HLA-A*03:01-restricted and HLA-A*11:01-restricted TCRs (Fig. 4a)^33^. Because of TCR4’s unique CDR3β length and superior cytolytic potency, we sought to characterize this receptor’s binding affinity, three-dimensional structure and fine specificity. For comparison, we used TCR3 as this receptor has a similar EC_50_ to TCR4 yet possesses more common CDR3 loop lengths.

**Extended Data Fig. 3:**
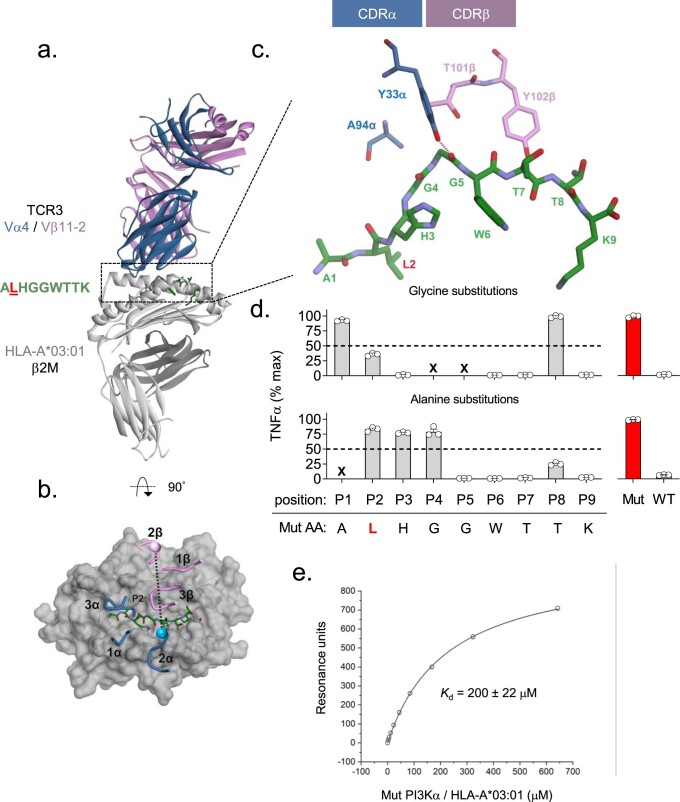
Structural details of the TCR3/pMut/HLA-A*03:01 complex, correlations with TCR3’s peptide recognition motif, and measurement of TCR3’s binding affinity. (**a**) Structural overview of the TCR3 pMut/HLA-A*03:01 ternary complex at 2.1 Å resolution. The color scheme is indicated and replicated throughout. (**b**) Top view of the pMut/HLA-A*03:01 complex displaying the crossing angle and positions of TCR3’s six CDR loops. Spheres show the centers of mass of the TCR Vα and Vβ domains. (**c**) Amino acids of TCR3’s CDRα and CDRβ loops that interact with the pMut peptide. The single hydrogen bond formed between TCR3 and pMut is indicated by a dashed red line. Amino acids are identified by standard one-letter codes followed by position number. The side chains of contacting residues from the TCR are also identified by AA and hemi-chain position number. (**d**) Identification of TCR3’s peptide recognition motif using alanine and glycine scanning. Intracellular FACS for TNFα production to Gly (upper) or Ala (lower) substituted peptides. TCR3-transduced T cells (identified by gating on mTCR^+^ lymphocytes) were co-cultured with HLA-A*03:01^+^ targets pulsed with 1μM of indicated peptides. Results shown as the mean ± SEM percent maximum response relative to the native pMut peptide using *n* = 3 biologic replicates per condition. ‘x’ indicates positions not amenable to substitution. (**e**) Steady-state binding equilibrium measuring the dissociation constant (*K*_d_) for TCR3 to the pMut/HLA-I complex. Results shown are the average ± standard deviation of *n* = 3 independent experiments.

We generated recombinant, soluble versions of both TCRs and measured their affinity toward the pMut/HLA-I complex using surface plasmon resonance (SPR). TCR4 has a *K*_d_ of 49 ± 11 μM (Fig. 4b,c), an affinity higher than many self-antigen-specific TCRs and within a range of pathogen^34^ and neoantigen-specific^35–37^ receptors. TCR3, by comparison, has a modestly lower affinity (*K*_d_ = 200 ± 22 μM) (Extended Data Fig. 3).

**Extended Data Fig. 4:**
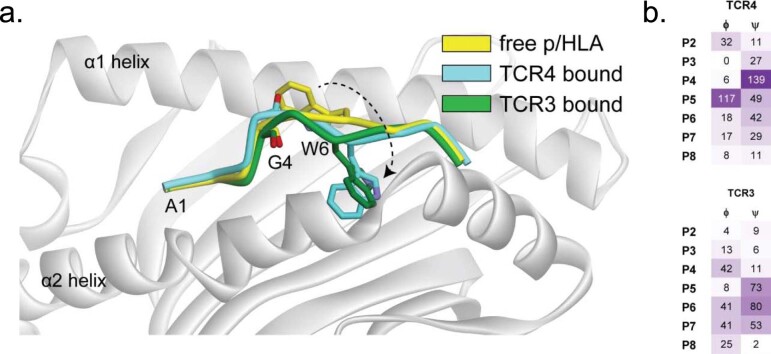
Mutant peptide conformational changes following TCR3 or TCR4 binding. (**a**) Visualization of the conformations of the pMut backbone in the unbound state and upon binding of TCR3 and TCR4, emphasizing changes that occur in the positions of the P6 Trp side chain and the P4 Gly backbone carbonyl. (**b**) Table summarizing changes (in degrees) in the backbone dihedral angles (ϕ) and (ψ) of the pMut peptide following binding of TCR4 (top) or TCR3 (bottom). For binding of TCR4, the peptide conformational change is primarily driven by a change in the P4 ψ and P5 ϕ, whereas for TCR3 the change is driven by smaller dihedral changes spanning P4 to P7.

**Extended Data Fig. 5:**
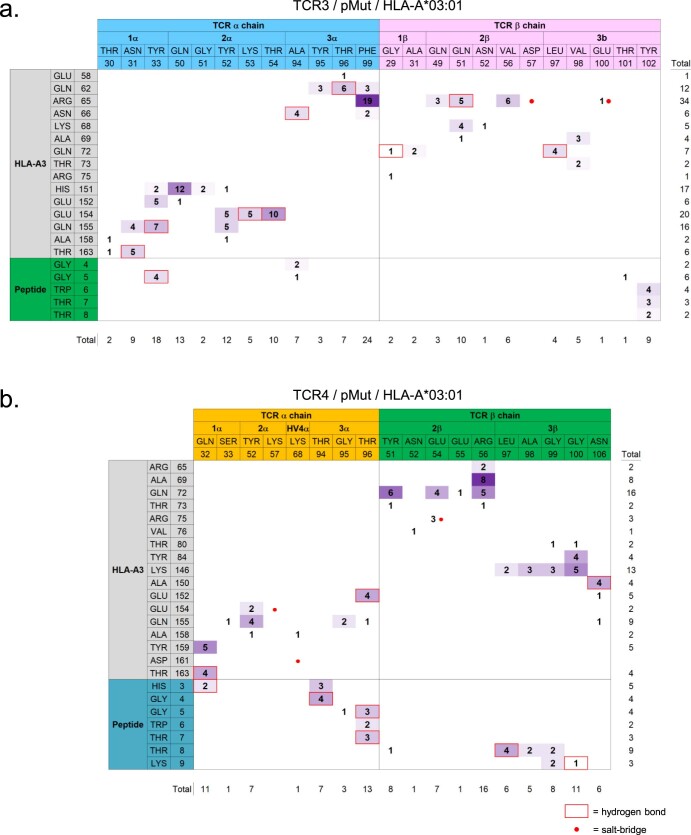
Contact matrices defining interatomic interactions between either TCR3 or TCR4 and the pMut/HLA-A*03:01 complex. Contact matrices for the (**a**) TCR3/pMut/HLA-A*03:01 or the (**b**) TCR4/pMut/HLA-A*03:01 complexes. Contacting amino acids, defined as interatomic distances ≤4 Å, are shown. The numbers in each cell represent the number of interatomic contacts for each amino acid pair listed. Red boxes indicate the presence of a hydrogen bond; red circles indicate a salt-bridge (can be >4 Å). Cells are colored according to the total number of contacts, from white (minimum) to purple (maximum). Residue numbers listed for each TCR hemichain and HLA-A*03:01 reflect subtraction of each protein’s leader sequence as indicated in the PDB data files.

We next crystallized and determined the X-ray structures of each TCR/pMut/HLA-A*03:01 ternary complex at resolutions of 3.1 Å and 2.1 Å, respectively (Fig. 4d, Extended Data Fig. 3a and Supplementary Table 2). Both TCRs dock using a conventional, diagonal orientation (Fig. 4e and Extended Data Fig. 3b)^32^. In most TCR structures, the two CDR3 loops focus solely on the central regions of the peptide^38^. This is not the case with TCR4. The CDR3α loop is positioned parallel to the core of the peptide backbone, extending from the P3 His to the P6 Trp (Fig. 4f). The unusually long CDR3β loop, on the other hand, reaches to the C-terminus of the peptide, forming hydrogen bonds with the side chain of the P8 Thr and the C-terminal carboxylate. By contrast, TCR3 engages only the central core of pMut (Extended Data Fig. 3c). It is noteworthy that both TCRs induce a major repositioning of the P6 Trp side chain upon binding (Extended Data Fig. 4). In this conformation, the residue becomes buried along the α_2_ helix of HLA-A*03:01 where it cannot be contacted by either receptor. We generated interatomic contact matrices to identify and quantify interactions between each TCR and the pMut/HLA-A*03:01 complex (Extended Data Fig. 5). Overall, TCR4 engages pMut with *n* = 30 intermolecular interactions, including *n* = 6 hydrogen bonds. TCR3, by contrast, engages pMut with far fewer contacts (*n* = 17) and only one hydrogen bond. Together, these data illustrate how the unique structural properties of TCR4, including its long CDR3β loop, permit the receptor to form an extended interface spanning nearly the entire length of pMut. By contrast, TCR3 engages pMut with more focused intermolecular interactions.

We next assessed the fine specificity of TCR4 and TCR3 using AA scanning to functionally define each receptor’s recognition motif (Fig. 4g and Extended Data Fig. 3d). TCR-transduced T cells were co-cultured with HLA-A*03:01^+^ target cells pulsed with Ala or Gly substituted peptides, and the effect of these changes on cytokine production was determined. Replacement with either AA at positions P5–P9 resulted in near complete loss of TCR4’s function. By contrast, TCR3 was either completely or partially tolerant to changes at P8, correlating with the receptor’s minimal contact to this region of pMut. Similarly, whereas alterations in P2 and P3 also led to loss of TCR4 function, TCR3 was permissive of substitutions at these sites.

To establish TCR4’s cross-reactivity potential, we selected residues that permitted reactivity when altered regardless of magnitude and identified TCR4’s peptide recognition motif as ‘x-x-x-x-G-W-T-T-K’ (‘x’ = any AA). Using the ScanProsite tool^39^, we surveyed the human proteome for sequences containing this motif. This search afforded two candidates: WT PI3Kα, as expected, and an unrelated peptide derived from transmembrane protein 87B (*TM87B*) (Supplementary Table 3). Notably, the TM87B-derived peptide failed to activate TCR4-transduced T cells (Fig. 4h). We conclude that TCR4 binds the pMut/HLA-I complex in a unique manner that maximizes CDR3 interactions across the length of pMut, thus contributing to its relatively high-affinity and low cross-reactivity profile.

### Therapeutic targeting of a *PIK3CA* public NeoAg

**Extended Data Fig. 6:**
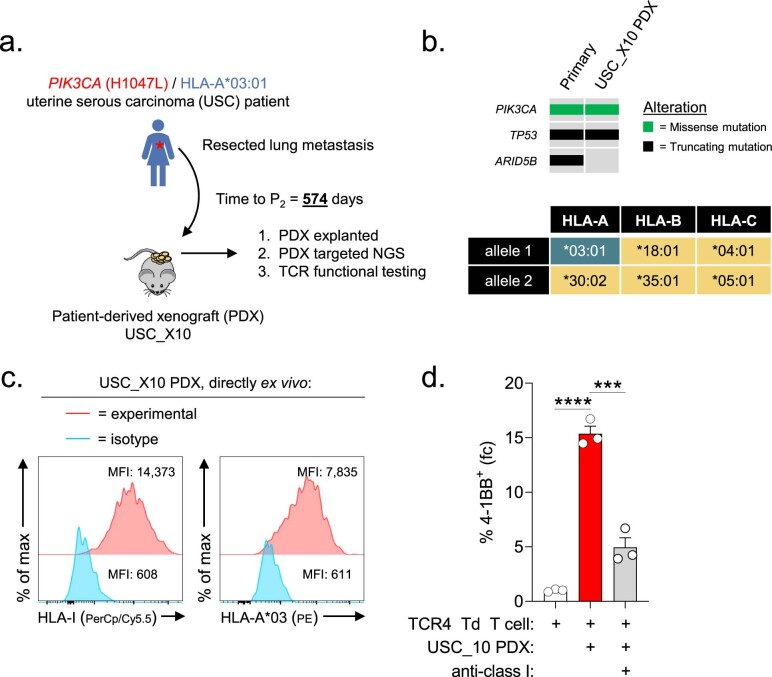
HLA class I-dependent recognition of an HLA-A*03:01^+^ / *PIK3CA* (H1047L)^+^ patient-derived xenograft by TCR4-transduced CD8^+^ T cells directly *ex vivo*. (**a**) Graphical overview of the generation and experimental testing of a patient-derived xenograft (PDX) from an HLA-A*03:01^+^ patient with uterine serous carcinoma (USC) associated with the *PIK3CA* (H1047L) mutation. The PDX (henceforth PDX USC_X10) was established from a surgically resected lung metastasis. (**b**) Comparison of the mutational landscape and HLA-I haplotype of the primary cancer versus PDX USC_X10 using high-coverage next-gen sequencing (NGS) with the MSK-IMPACT platform. The clonal *PIK3CA* (H1047L) mutation and *TP53* mutation were shared; however, a subclonal *ARID5B* (p.L1124fs) mutation (variant allele frequency < 7%) detected in the primary tumor was not found in the lung metastasis-derived PDX. (**c**) FACS analysis of HLA-I and HLA-A*03 expression on a single-cell digest of the explanted USC_X10 PDX after two serial passages in NSG mice. (**d**) Comparison of the fold-change (fc) in 4-1BB expression on TCR4 transduced CD8^+^ T cells co-cultured overnight with a single cell-digest of explanted PDX USC_X10 cells in the absence or presence of an anti-HLA-I antibody. Results shown as mean ± SEM using *n* = 3 biologic replicates per condition. ****P < 0.0001 and ***P = 0.001 using a two-sided Student’s t-test with Bonferroni correction.

We next established TCR4’s capacity to therapeutically engage cancer cells. First, we measured the response of TCR4-transduced T cells to a patient-derived xenograft (PDX) generated from an HLA-A*03:01^+^ patient with Mut *PIK3CA* uterine serous carcinoma (PDX USC_X10) (Extended Data Fig. 6a). Next-generation sequencing (NGS) and fluorescence-activated cell sorting (FACS) analysis confirmed that the mutational landscape and HLA-I haplotype of the PDX matched the patient’s primary tumor except for a subclonal *ARID5B* mutation (Extended Data Fig. 6b,c). To quantify T cell recognition of tumor cells, we measured changes in 4-1BB expression. Compared to TCR4-transduced T cells alone, co-culture with the PDX resulted in a 15.3 ± 0.7-fold increase in 4-1BB (Extended Data Fig. 6d). This change was significantly blocked by an anti-HLA-I antibody (*P* < 0.001).

**Extended Data Fig. 7:**
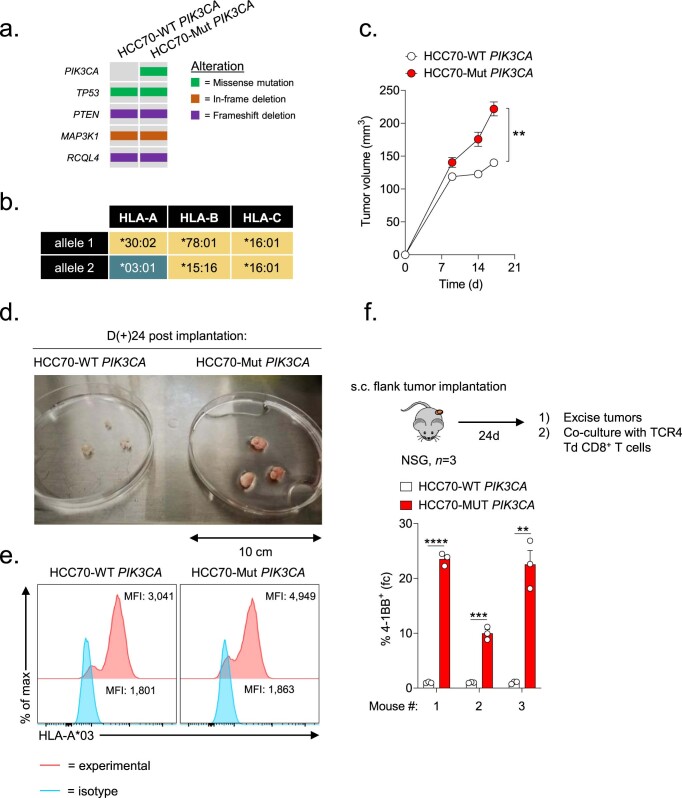
Establishment of a transplantable HLA-A*03:01^+^ / *PIK3CA* (H1047L) breast cancer tumor model and associated WT *PIK3CA* control. (**a**) Mutational landscape and (**b**) HLA-I haplotype of isogenic HCC70 breast adenocarcinoma tumor cell lines that express either WT *PIK3CA* (HCC70-WT *PIK3CA*) or *PIK3CA* (H1047L) (HCC70-Mut *PIK3CA*). (**c**) In vivo growth kinetics of HCC70-WT *PIK3CA* or HCC70-Mut *PIK3CA* following subcutaneous (s.c.) implantation of 3 x 10^6^ tumor cells into immune-deficient NSG mice. Data shown as mean ± SEM for *n* = 3 mice per tumor cell line. **P = 0.0056 using a two-way ANOVA. (**d**) Image comparing explanted tumor sizes and (**e**) FACS analysis of HLA-A*03 expression on HCC70-WT *PIK3CA* or HCC70-Mut *PIK3CA* cells 24 days after s.c. implantation. (**f**) Comparison of the fold-change (fc) in 4-1BB expression on TCR4 transduced (Td) CD8^+^ T cells co-cultured overnight with a single cell-digest of explanted HCC70-WT *PIK3CA* or HCC70-Mut *PIK3CA* tumor cells. Results shown as mean ± SEM using *n* = 3 biologic replicates per condition. ****P < 0.0001, ***P = 0.0002, and **P = 0.001 using a two-sided Student’s t-test.

The prolonged passage time of PDX USC_X10 precluded its use for in vivo therapeutic experiments. Therefore, we developed an isogenic tumor model using HCC70, a breast adenocarcinoma cell line that expresses HLA-A*03:01 (Extended Data Fig. 7a,b). HCC70 harboring WT *PIK3CA* (HCC70-WT *PIK3CA*) and *PIK3CA* (H1047L) (HCC70-Mut *PIK3CA*) efficiently engrafted in immune-deficient NOD scid gamma (NSG) mice. However, the growth rate of HCC70-Mut *PIK3CA* was significantly greater than its WT counterpart (Extended Data Fig. 7c,d), a finding consistent with Mut *PIK3CA*’s function as a driver oncogene. Explanted HCC70-Mut *PIK3CA*, but not HCC70-WT *PIK3CA*, triggered 4-1BB upregulation on TCR4-transduced T cells ex vivo (Extended Data Fig. 7e,f).

**Fig. 5:**
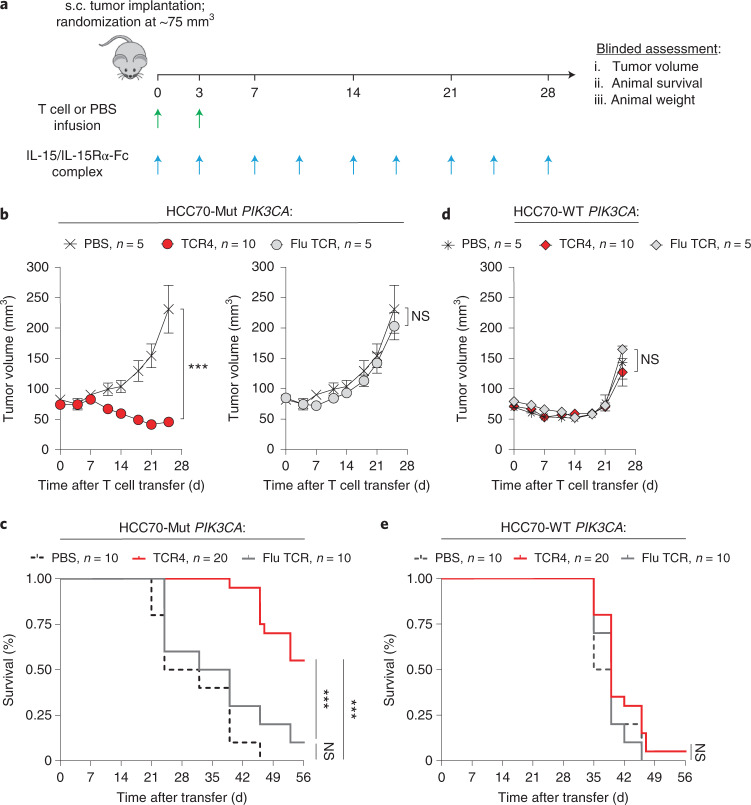
In vivo anti-tumor efficacy of adoptively transferred T cells genetically engineered with a *PIK3CA* public NeoAg TCR. **a**, Experimental overview for the evaluation of the in vivo anti-tumor efficacy and safety of targeting Mut *PIK3CA* using TCR-transduced CD8^+^ T cells. Mice were randomized between indicated treatment groups once subcutaneously (s.c.) implanted HCC70-Mut *PIK3CA* or HCC70-WT *PIK3CA* tumors were established to ~75 mm^3^. All mice received a twice-weekly intraperitoneal injection of 1 μg of IL-15 pre-complexed with IL-15Rα-Fc (1:1 M) and intravenous injection of CD8^+^ T cells transduced with TCR4, CD8^+^ T cells transduced with an influenza (Flu)-specific TCR, or PBS. Mice received 7.5 × 10^6^ and 2.5 × 10^6^ TCR^+^ T cells on D0 and D3 after randomization. Tumor volumes (**b**) and overall survival (**c**) of mice bearing HCC70-Mut *PIK3CA* tumors infused with the indicated treatment. Tumor volumes (**d**) and overall survival (**e**) of mice bearing established HCC70-WT *PIK3CA* tumors infused with the indicated treatment. Data in **b** and **d** are shown as mean ± s.e.m. and are representative of two independently performed experiments (TCR4 *n* = 10, Flu TCR *n* = 5, PBS *n* = 5). ***P < 0.001 and NS using two-way ANOVA. Pooled survival data from two identically performed experiments are shown in **c** and **e** and are plotted as a Kaplan–Meier survival curve (TCR4 *n* = 20, Flu TCR *n* = 10, PBS *n* = 10). ***P < 0.001 and NS using log-rank test. D, day; NS, not significant.

Using this model, we tested whether adoptive cell transfer (ACT) of TCR4-transduced CD8^+^ T cells could mediate regression of established HCC70 tumors without causing off-target effects. For these experiments, we used an HLA-A*03:01-restricted influenza nucleoprotein (Flu) TCR as a specificity control (Supplementary Fig. 9). After HCC70-WT *PIK3CA* or HCC70-Mut *PIK3CA* tumors grew to ~75 mm^3^, mice were randomized to receive by intravenous injection one of the following treatments: (1) TCR4-transduced CD8^+^ T cells, (2) CD8^+^ T cells transduced with the Flu-specific TCR or (3) a PBS control (Fig. 5a). All mice received an extended half-life variant of IL-15 to mimic the physiologic effect of host lymphodepletion^40^. Mice with HCC70-Mut *PIK3CA* tumors and treated with TCR4 had a significant reduction in tumor volumes and enhanced survival compared to mice that received the Flu-specific TCR or PBS controls (Fig. 5b,c and Supplementary Fig. 10a). By contrast, no significant differences were observed in tumor growth or survival between treatment arms in mice with HCC70-WT *PIK3CA* tumors (Fig. 5d,e and Supplementary Fig. 10b). This latter finding suggests that TCR4 did not mediate an off-target effect on cells expressing WT *PIK3CA*. None of the TCR-treated mice experienced a significant loss of body weight compared to PBS controls (Supplementary Fig. 11). We conclude that ACT of TCR4-transduced T cells safely mediates Mut-specific anti-tumor responses against established tumors in vivo.

### Clonality and immunogenicity of a *PIK3CA* public NeoAg

**Fig. 6:**
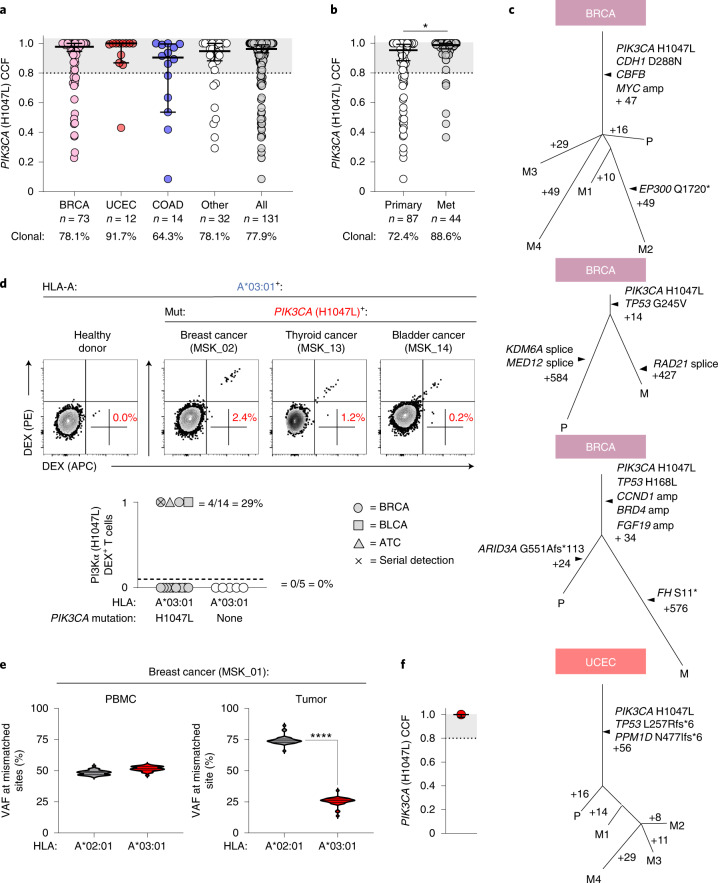
Clonality, immunogenicity and immune resistance to a *PIK3CA* public NeoAg in patients with cancer. **a**, Pan-cancer analysis measuring the clonality of *PIK3CA* (H1047L) in *n* = 131 unique MSK patients with cancer. Clonality is defined as a CCF of ≥80%, indicated by the shaded gray area. **b**, Comparison of *PIK3CA* (H1047L) CCF in primary versus metastatic tumor sites. *P* = 0.0245 using two-sided Student’s *t*-test. **c**, Phylogenetic analysis measuring the clonal conservation of *PIK3CA* (H1047L) in primary (P) and metastatic (M) tumor sites within the same patient. **d**, Representative FACS and summary plot for the detection of circulating CD8^+^ T cells specific for the pMut/HLA-A*03:01 (A*03:01) epitope in *n* = 14 HLA-A*03:01^+^ patients with a history of a *PIK3CA* (H1047L) cancer or *n* = 5 HLA-A*03:01^+^ healthy donors. Percentages in FACS plots represent the frequency of gated live^+^CD8^+^dual pMut/HLA-A*03:01 dextramer^+^ lymphocytes. 0 = no detection; 1 = detection. **e**, **f**, Variant allele frequency (VAF) at each of the positions in *HLA-A* that are mismatched between HLA-A*02:01 and HLA-A*03:01 are shown for the PMBC and tumor samples of patient MSK_01 who demonstrated a tumor-specific HLA-A*03:01 LOH in the setting of *PIK3CA* (H1047L) clonal conservation. *P* < 0.0001 by two-sided Student’s *t*-test. Results in **a**, **b** and **f** are displayed as median ± 95% confidence interval. Violin distributions in **e** are centered around the median (solid horizontal line) with quartile ranges displayed above and below (dashed horizontal lines). The maxima and minima are represented by the top and bottom of each plot. ATC, anaplastic thyroid cancer; BLCA, bladder cancer; BRCA, breast invasive carcinoma; COAD, colon adenocarcinoma; DEX, HLA dextramer; UCEC, uterine corpus endometrial carcinoma.

NeoAg clonal heterogeneity is a major resistance mechanism to T-cell-based immunotherapies^4–6^. Because *PIK3CA* gain-of-function mutations are critical to cancer cell fitness^41,42^, we hypothesized that Mut *PIK3CA* would be clonally conserved across patients and disease sites. To test this hypothesis, we determined the cancer cell fraction (CCF), a measurement of mutation clonality, in a pan-cancer cohort of *n* = 131 patient samples harboring *PIK3CA* (H1047L). We discovered that Mut *PIK3CA* was clonally expressed in most tumors (*n* = 102/131; 77.9%) (Fig. 6a)_._ Critically, Mut *PIK3CA* clonality was observed in both primary and metastatic sites (Fig. 6b). Phylogenetic analysis using matched biospecimens revealed clonal preservation of Mut *PIK3CA* in the face of branched evolution for other mutated genes (Fig. 6c).

**Extended Data Fig. 8:**
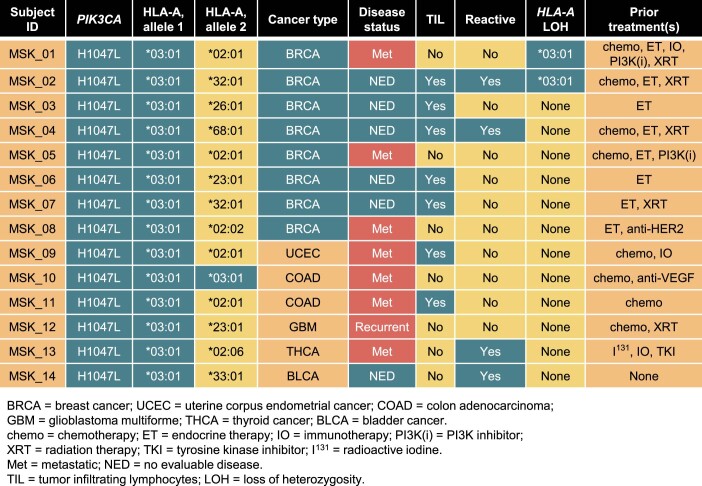
Demographics of HLA-A*03:01^+^ cancer patients expressing the *PIK3CA* (H1047L) public neoantigen.

**Extended Data Fig. 9:**
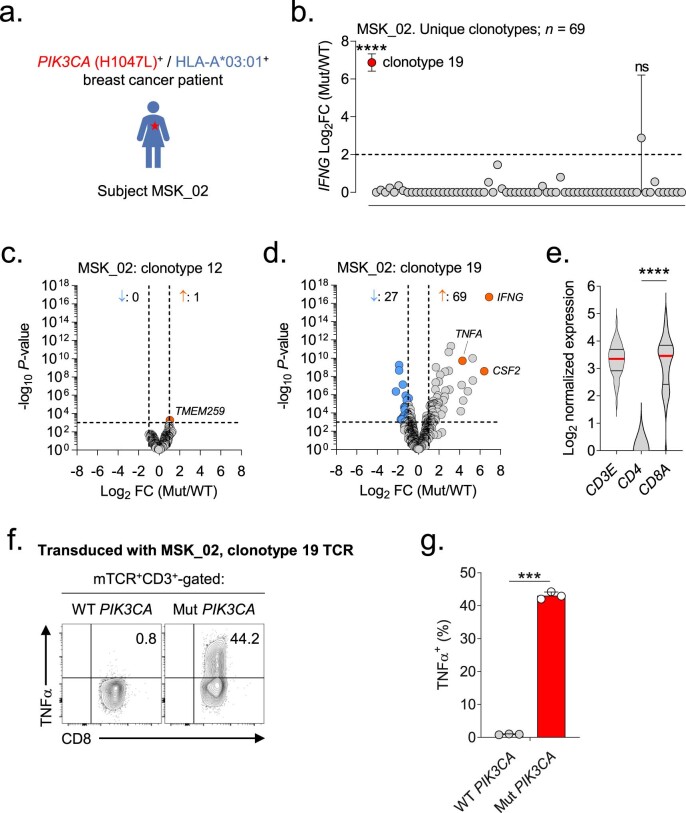
Application of the SIFT-seq discovery platform for the identification and retrieval of a Mut *PIK3CA*-specific TCR from a public NeoAg-expressing breast cancer patient. (**a**) PBMC was sampled from an HLA-A*03:01^+^ breast cancer patient (subject MSK_02) whose tumor harbored the *PIK3CA* (H1047L) mutation (Mut). (**b**) Log_2_ fold-change (FC) ratio of *IFNG* transcripts from *n* = 69 unique TCR clonotypes identified using single-cell RNA and V(D)J TCR sequencing from a screen positive ‘hit’ well. Matched aliquots of sensitized T cells from the patient were stimulated with Mut *PIK3CA* or wildtype *PIK3CA* (WT) prior to single-cell sequencing. The mean *IFNG* ratio for all evaluable TCR clonotypes is shown. Statistical analyses were performed for all clonotypes with a minimal ratio ≥2 (dashed line). ****P = 1.98e^−17^ and ns = not significant using a two-sided Welch’s t-test. Volcano plots displaying global transcriptomic changes for (**c**) non-reactive MSK_02 clonotype 12 or (**d**) reactive MSK_02 clonotype 19 following stimulation with Mut versus WT *PIK3CA*. Vertical and horizontal dashed lines indicate thresholds for gene expression FC and statistical significance, respectively. Orange and blue dots represent significantly up and down-regulated genes following Mut *PIK3CA* stimulation, respectively. (**e**) Violin plots depicting transcript levels for the lineage markers *CD3E*, *CD4*, and *CD8A* from MSK_02 clonotype 19. Violin distributions are centered around the median (red horizontal line) with quartiles ranges displayed above and below (dashed horizontal lines). The maxima and minima are represented by the top and bottom of each plot. ****P < 0.0001 using a two-sided Student’s t-test with Bonferroni correction. (**f**) Representative FACS plots and (**g**) summary bar graph of TNFα production by CD8^+^ T cells transduced with the SIFT-seq retrieved MSK_02 clonotype 19 TCR. Transduced T cells were co-cultured with HLA-A*03:01^+^ target cells that express either WT or Mut *PIK3CA*. Symbols and bar graphs displayed as mean ± SEM using *n* = 3 biologic replicates per condition. ***P = 0.0003 using a two-sided Student’s t-test.

To establish whether the *PIK3CA* public NeoAg is spontaneously immunogenic, we collected PBMC samples from *n* = 14 HLA-A*03:01^+^ patients with a history of a Mut *PIK3CA* cancer identified using MSK-Integrated Mutation Profiling of Actionable Cancer Targets (IMPACT) (Extended Data Fig. 8 and Supplementary Fig. 12)^43–45^. We analyzed patient PBMCs directly ex vivo and after IVS using dual fluorochrome-labeled HLA-A*03:01 multimers loaded with pMut. As controls, we analyzed PBMCs from *n* = 5 HLA-A*03:01^+^ HDs using identical stimulation conditions. We detected *PIK3CA* public NeoAg-specific T cells in four of 14 (29%) patients and zero of five (0%) HDs (Fig. 6d). Application of the SIFT-seq platform to PBMCs from patient MSK_02 successfully retrieved a patient-derived TCR that confers reactivity to target cells that express Mut, but not WT, *PIK3CA* (Extended Data Fig. 9). In patient MSK_04, we detected *PIK3CA* public NeoAg-specific T cells in serially collected samples, indicating a sustained response to this public NeoAg. Together, these data indicate that the *PIK3CA* public NeoAg is immunogenic and capable of driving T cell clonal expansion in vivo in a subset of cancer patients.

**Extended Data Fig. 10:**
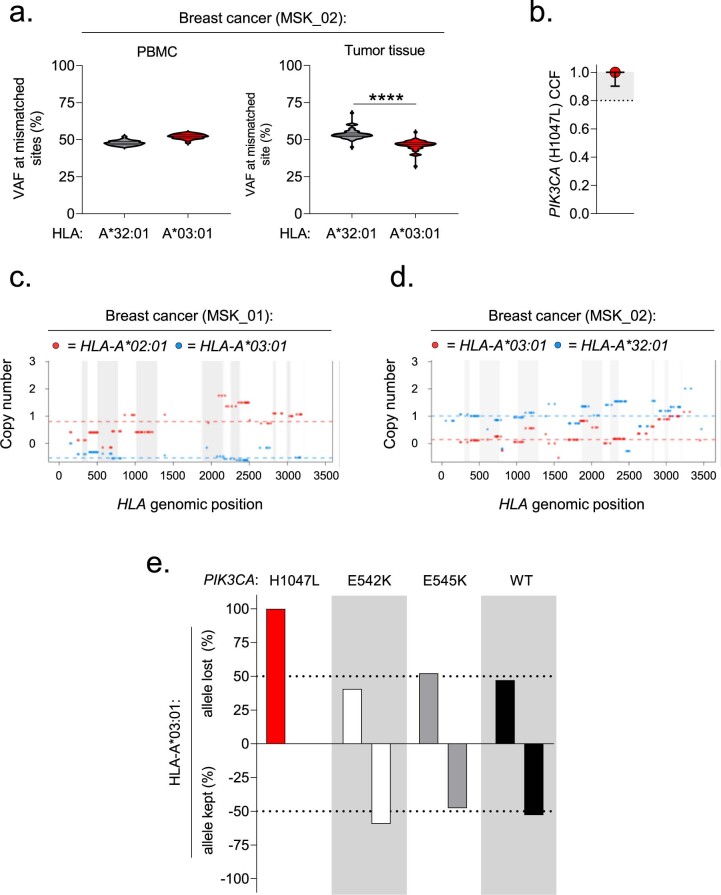
*HLA-A* loss of heterozygosity events in HLA-A*03:01^+^ cancer patients with tumors harboring *PIK3CA* (H1047L), *PIK3CA* (E542K), *PIK3CA* (E545K), or WT *PIK3CA*. (**a**) Variant allele frequency (VAF) at each of the positions in *HLA-A* that are mismatched between HLA-A*32:01 and HLA-A*03:01 are shown for the PMBC and tumor samples of subject MSK_02. Violin distributions are centered around the median (solid horizontal line) with quartiles ranges displayed above and below (dashed horizontal lines). The maxima and minima are represented by the top and bottom of each plot. ****P < 0.0001 using a two-sided Student’s t-test. (**b**) Cancer cell fraction (CCF) for *PIK3CA* (H1047L) in subject MSK_02. Results displayed as median ± 95% confidence interval. **(c,d)** Display of *HLA-A* allele-specific copy numbers for subjects MSK_01 and MSK_02 as an alternative method for establishing *HLA* loss of heterozygosity. Results were generated using the LOHHLA algorithm. (**e**) Comparison of which *HLA-A* allele is lost in HLA-A*03:01^+^ cancer patients with an *HLA-A* LOH event and a tumor (any histology) harboring *PIK3CA* (H1047L), *PIK3CA* (E542K), or *PIK3CA* (E545K) mutation. *HLA-A* LOH events in HLA-A*03:01^+^ patients with a WT *PIK3CA* breast cancer are shown as an additional comparison. Hashed lines indicate ±50% threshold.

Immunogenic NeoAgs apply evolutionary pressure to cancer cells, selecting for populations with resistance to immune recognition^46^. Resistance can occur through multiple mechanisms^47^, including allele-specific HLA loss of heterozygosity (LOH)^48,49^. We sought to determine whether *PIK3CA* public NeoAg-expressing tumors exhibited evidence of cancer cell-intrinsic resistance to immunologic recognition. Targeted exome sequencing did not reveal loss-of-function mutations in genes associated with resistance to T cell immunotherapies^50,51^, including *TAP1/2*, *CALR*, *B2M*, *HLA-A*, *JAK1/2* and *IFNGR1*. By contrast, NGS data from *n* = 31 Mut *PIK3CA*^+^/HLA-A*03:01^+^ patients revealed HLA-A LOH involving the HLA-A*03:01 allele in two patients (Fig. 6e and Extended Data Fig. 10a–d), whereas no patients exhibited LOH involving the non-HLA-A*03:01 allele. Both patients maintained clonal expression of Mut *PIK3CA* (Fig. 6f and Extended Data Fig. 10b). As controls, we measured the proportion of *HLA-A* LOH events involving HLA-A*03:01 versus the alternative *HLA-A* allele in cancers with *PIK3CA* (E542K) or *PIK3CA* (E545K) (Extended Data Fig. 10e). Because neither of these *PIK3CA* mutations generate an HLA-A*03:01-restricted neoepitope (Extended Data Fig. 2), we hypothesized that *HLA-A* LOH events would occur at random. Among HLA-A*03:01^+^ patients with *PIK3CA* (E542K) and an *HLA-A* LOH event (*n* = 32), 13 of 32 (40.6%) lost HLA-A*03:01, whereas 19 of 32 (59.4%) lost the alternative *HLA-A* allele. Similarly, of *n* = 44 HLA-A*03:01^+^ patients with a *PIK3CA* (E545K) cancer and an *HLA-A* LOH event, 23 of 44 (52.2%) lost HLA-A*03:01, whereas 21 of 44 (47.7%) lost an alternative *HLA-A* allele. Finally, we measured the proportion of *HLA-A* LOH events in HLA-A*03:01^+^/WT *PIK3CA* breast cancers. Here also, of *n* = 210 *HLA-A* LOH events, 99 of 210 (47.1%) involved HLA-A*03:01, whereas 111 of 210 (52.9%) involved the alternative *HLA-A* allele. These findings suggest an unbiased loss of one *HLA-A* allele over another in cases where a cancer is incapable of generating an HLA-A*03:01-restricted *PIK3CA* neoepitope. We conclude that the *PIK3CA* public NeoAg is typically clonal, immunogenic in patients with cancer and, in select cases, correlated with a mechanism of neoepitope-specific immune resistance.

## Discussion

Here we demonstrate that Mut *PIK3CA*, among the most common genomically altered driver genes^18,19^, is immunogenic in the context of a prevalent HLA-I allele, thereby creating a public NeoAg. Mechanistically, we determined that the molecular basis for *PIK3CA* public NeoAg immunogenicity results from the creation of an optimal HLA-I anchor residue. Unlike private NeoAgs, we discovered that the *PIK3CA* public NeoAg is shared among patients and frequently clonally conserved. Using SIFT-seq, we generated with high efficiency a panel of TCR gene sequences that confer specificity to this shared neopeptide from both HDs and a patient with cancer (Supplementary Table 4). Several recent reports have described alternative methods for retrieving NeoAg-specific TCRs^52–56^. SIFT-seq uniquely incorporates six features that are relevant for TCR candidate development (Supplementary Table 5), including establishing that a putative neopeptide is endogenously processed and presented. The importance of this latter attribute is highlighted by the following: although HLA-binding algorithms correctly anticipate the ALHGGWTTK neopeptide identified in our studies, more than 88% of predicted HLA-A*03:01-restricted high-affinity peptides derived from full-length Mut or WT *PIK3CA* were not detected by HLA-IP LC–MS/MS (Extended Data Fig. 2 and Supplementary Fig. 6). TCR clonotypes with desirable specificity and binding attributes are expected to be rare. Screening strategies that focus exclusively on bona fide neoepitopes increases the likelihood of retrieving clonotypes with diverse and potentially unique attributes.

Stratification of our TCR panel members revealed that TCR4 is co-receptor-independent, functionally avid and capable of sustained cytolysis of Mut *PIK3CA*^+^/HLA-A*03:01^+^ target cells. Comparison of TCR4’s gene sequence to more than 17,000 TCRs restricted by HLA-A*03:01 and HLA-A*11:01 revealed that this receptor possesses an unusually long CDR3β loop. The functional significance of this feature was uncovered by resolving the structure of this receptor in complex with pMut/HLA-I. The CDR3 loops of most TCRs, including TCR3, engage the central core (P4–P7) of an HLA-I-bound peptide^31,38^. However, the *PIK3CA* pMut/HLA-I complex presents a challenge: the surface it displays to a TCR is relatively ‘bland’ because it is dominated by the peptide’s backbone rather than exposed side chains typically associated with peptide immunogenicity^57^. Indeed, upon both TCR3 and TCR4 binding, the side chain of the neopeptide’s most distinctive residue (P6 Trp) becomes fully buried in the HLA-A*03:01 peptide-binding groove, further increasing its largely featureless nature. Recent efforts to rationalize the importance of bulky, multifunctional AAs such as Trp in peptide immunogenicity have emphasized their importance in forming key TCR contacts^37,57^. The recognition of the *PIK3CA* public NeoAg by TCR3 and TCR4 defies such expectations.

TCR4’s unusually long CDR3β loop enables the receptor to form an extended and highly complementary interaction with the peptide. This unusual configuration likely contributes not only to TCR4’s affinity but also to its specificity as the receptor was found to lack measurable cross-reactivity to alternative epitopes derived from the human proteome. The exquisite specificity of TCR4, combined with (1) the tumor-specific expression of Mut *PIK3CA* and (2) the absence of a stable pWT/HLA complex on normal tissues, establishes a favorable safety profile for this receptor’s potential as a clinical candidate. Indeed, in proof-of-concept experiments, ACT of T cells genetically engineered with TCR4 caused mutation-specific tumor regression in the absence of measurable off-target effects.

Circulating T cells specific for the *PIK3CA* public NeoAg were detected in ~30% of HLA-A*03:01^+^/Mut *PIK3CA*^+^ patients with cancer. LOH for the restricting HLA-I allele of a NeoAg is a precise means of immune-editing that permits cancer cells to retain expression of a Mut driver allele^48,49^. We found selective LOH for HLA-A*03:01, but not alternative *HLA-A* alleles, in two of 31 (7%) patients. By contrast, in HLA-A*03:01^+^ patients with cancers harboring either WT *PIK3CA* or a *PIK3CA* mutation that does not generate a public NeoAg, *HLA-A* LOH events were randomly divided between HLA-A*03:01 and the alternative *HLA-A* allele. Similar findings were recently reported using an independent pan-cancer dataset of more than 83,000 HLA-I-typed patients^49^. Although these observations will require additional experiments to establish causality, they suggest that the immunogenicity of *PIK3CA* public NeoAg might lead to acquired immune-escape while preserving oncogenic driver expression in a subset of patients.

Across our cohort of public NeoAg-expressing cancers, targeted exome sequencing did not reveal alternative genomic alterations that might compromise antigen processing and presentation. However, this finding does not preclude tumor cell-intrinsic transcriptional and/or epigenetic mechanisms of antigen presentation immune-escape, which have also recently been reported^46,58^. We have assembled and studied what is, to our knowledge, the largest collection of biospecimens from patients who express an identical NeoAg resulting from an NSSM. Nevertheless, a limitation of our study is that the sample size remains underpowered to conclusively establish correlates among patient demographics, tumor-specific parameters and immunogenicity. Finally, although we demonstrated that Mut *PIK3CA* is typically clonal, gain-of-function mutations in *PIK3CA* often occur as a late evolutionary event during tumorigenesis^17^. Consequently, it is possible that a WT *PIK3CA* cancer may recur despite an effective T cell response targeting the *PIK3CA* public NeoAg.

Although we focused on Mut *PIK3CA*, the modular nature of the SIFT-seq platform might readily be applied to the discovery of public NeoAg-specific TCRs resulting from the more than 200 additional driver genes identified to date^18^. Recent regulatory approvals have been made for tissue-agnostic therapies targeting specific genomic alterations identified using clinical NGS, including NTRK fusion inhibitors^59^ and immune checkpoint inhibitors in microsatellite-unstable cancers^60^. An analogous approach might be applied for the future clinical development of target-specific but tissue-agnostic TCR-based therapies targeting public NeoAgs^47^, including the *PIK3CA* public NeoAg described here.

## Methods

### Primary cells and cell lines

Leukopaks from HDs were purchased from the New York Blood Center. HLA-typed leukoreduction system chambers from HDs were obtained from the Stanford Blood Center. PBMCs were isolated by density-gradient centrifugation using lymphocyte separation medium (Corning) and cryopreserved until ready for use. High-resolution genomic *HLA* typing for HD PBMCs was performed by HistoGenetics. The retroviral packaging line 293GP was purchased from Takara Bio (cat. no. 631458), and the HCC70 cell line was purchased from the American Type Culture Collection (cat. no. CRL-2315). Isogenic HCC70 cell lines stably expressing either WT *PIK3CA* or Mut *PIK3CA* were obtained by RV infection, followed by subcloning. COS-7 cells were obtained through a material transfer agreement (MTA) from S. A. Rosenberg (National Cancer Institute). Human primary cells were cultured in complete RPMI 1640 (Gibco) media supplemented with antibiotics and 10% human AB serum (GeminiBio). Cell lines were cultured and maintained in RPMI 1640 (Gibco) media supplemented with Pen–Strep (Gibco), gentamycin (MP Biomedicals) and 10% FBS (GeminiBio).

### PBMC collection from *PIK3CA* public NeoAg-expressing cancer patients

All patients provided written informed consent for tumor and white blood cell sequencing and review of patient medical records for demographic, pathological and treatment information under a Memorial Sloan Kettering Cancer Center (MSKCC) institutional review board-approved biospecimen umbrella protocol (protocol 12-245; ClinicalTrials.gov ID: NCT01775072). PBMCs were collected in CPT tubes (BD Biosciences) and processed according to the manufacturer’s instructions. Candidate patients were identified based on results from the MSK-IMPACT clinical NGS platform^43,44^. DARWIN, an automated genotype-driven enrollment tool that matches NGS results with upcoming patient clinic visits^45^, was used to identify *PIK3CA* (H1047L)^+^/HLA-A*03:01^+^ patients for biospecimen collection.

### Plasmids and peptides

All *PIK3CA* and *HLA* gene sequences were synthesized and cloned into the pcDNA3.1^+^ vector (GenScript). Constructs encoding full-length and overlapping minigenes of WT *PIK3CA*, individual *PIK3CA* hotspot mutations (E542K, E545K and H1047L/R*)* and HLA-A*03:01, HLA-A*03:02, HLA-A*11:01, HLA-A*69:01, HLA-B*35:03, HLA-B*49:01, HLA-C*04:01 and HLA-C*07:01 were generated. Plasmid vectors were linearized using XbaI (New England Biolabs) and purified using the QIAquick PCR Purification Kit (Qiagen) following manufacturer instructions. In vitro transcribed RNA was generated using the HiScribe T7 High Yield RNA Synthesis Kit (New England Biolabs) following the manufacturer’s instructions. TCR α/β was synthesized and cloned into the pMSGV-1 RV plasmid, as previously described^61^. The human variable regions of retrieved TCRs were fused to a modified murine constant chain (mTCR) to facilitate identification of transduced T cells and promote proper chain pairing^62^. HPLC grade 9-mer peptides (>99%) for pWT, pMut, alanine and glycine substituted variants of pMut and a peptide derived from TM87B were manufactured by GenScript.

### IVS of HD and patient PBMCs

PBMCs were plated in tissue culture flasks at 1 × 10^6^ cells per cm^2^ in complete media in the absence of cytokine for 2 hours at 37 °C to separate the adherent (monocyte-containing) and non-adherent (T-cell-containing) fractions. T cells were enriched from the non-adherent fraction by untouched negative selection (STEMCELL Technologies). To generate moDCs, the adherent fraction was washed with PBS, and fresh complete media supplemented with recombinant human IL-4 and GM-CSF (400 IU ml^−1^) were provided every alternate day. moDCs were stimulated with LPS (Invitrogen) and IFN-γ (Miltenyi Biotec) for 16–24 hours before transfection. moDCs were electroporated with 100 μg ml^−1^ of mRNA encoding an individual *PIK3CA* hotspot mutation using the Neon Transfection system (10-μl tip, setting: 1,325 V/10 ms/3 pulses). HD T cells were co-cultured with electroporated moDCs at an effector-to-target (E:T) ratio of 3:1 in the presence of IL-21 (30 ng ml^−1^) in 24-well non-TC plates (Falcon). Wells were supplemented with fresh media containing IL-7 and IL-15 (10 ng ml^−1^ each) every 3 days during the duration of the in vitro culture. All cytokines were purchased from Miltenyi Biotec. For patient-derived samples, PBMCs were enriched for CD8^+^ T cells and stimulated with autologous monocytes pulsed with pMut (1 μg ml^−1^) in the presence of 300 IU ml^−1^ of IL-2.

### Mutation-specific qPCR screen

Aliquots containing 2 × 10^4^ cells from individual parent IVS wells were harvested, split equally into two daughter wells and stimulated with moDCs electroporated with mRNA encoding WT or Mut *PIK3CA* for 3 hours at an E:T ratio of 1:2. After stimulation, total mRNA was purified (Qiagen), transcribed into cDNA (ABI), and levels of *IFNG* transcript were quantified by real-time qPCR (ABI QuantStudio 5) using the TaqMan Fast Advanced MasterMix (ABI). Delta cycle threshold (Ct) values for matched Mut versus WT stimulated wells were calculated. Wells with a positive delta Ct value of ≥2 s.d. from the mean were selected for downstream single-cell immune-profiling using SIFT-seq.

### Single-cell immune-profiling

Single-cell RNA and V(D)J sequencing was performed using the 10x Genomics platform. One × 10^5^ cells from a qPCR-positive well were harvested and co-cultured with moDCs electroporated with mRNA encoding WT or Mut *PIK3CA* at an E:T ratio of 1:10 for 3 hours. Final cell concentration was determined by cell counting on a hemocytometer, and cell concentration was adjusted to approximately 700 to 1,200 cells per microliter to maximize the likelihood of achieving the desired cell recovery target, with an initial cell viability of more than 90%. The single-cell suspension was mixed with RT Master Mix and loaded together with barcoded single-cell 5′ gel beads and partitioning oil onto Single Cell A Chip to generate gel beads in emulsion (GEMs) using Chromium Controller. Cell lysis and barcoded reverse transcription of RNAs from single cells were finished inside each GEM. Barcoded cDNA product was recovered through post GEM-RT cleanup and PCR amplification. cDNA quality control and quantification were determined by High Sensitivity D5000 DNA ScreenTape analysis (Agilent Technologies) and Qubit dsDNA HS Assay Kit (Thermo Fisher Scientific). Fifty nanograms of cDNA was used for 5′ gene expression library construction, and each sample was indexed by a Chromium i7 Sample Index Kit, which was run on an Illumina HiSeq 4000 sequencer with 2 × 100 base pairs (bp) paired reads to achieve at least 30,000 read pairs per cell. Full-length V, D and J gene segments were amplified using a Chromium Single Cell V(D)J Enrichment Kit (human T cells) to generate enrichment products. Enriched product was measured by D5000 DNA ScreenTape analysis and Qubit dsDNA HS Assay Kit, and then 50 ng of enrichment TCR product was used for library construction. Single Cell V(D)J enriched libraries were sequenced on HiSeq 4000 to produce paired 2 × 150-bp reads at 5,000 read pairs per cell. The raw single-cell RNA sequencing and VDJ data were pre-processed (demultiplexing of cellular barcodes, read alignment and generation of feature-barcode matrix) using Cell Ranger (10x Genomics, version 2.1.1) with the annotation files ‘vdj_GRCh38_alts_ensembl-3.1.0-3.1.0’ and ‘GRCh38-3.0.0’ for the V(D)J sequencing and gene expression libraries, respectively. Detailed quality control metrics were generated and evaluated using single-cell analysis packages from Bioconductor, including DropletUtils, scater and scran. Genes detected in fewer than three cells and cells where fewer than 200 genes had non-zero counts were filtered out and excluded from subsequent analysis. Low-quality cells with more than 15% of the read counts derived from the mitochondrial genome were also discarded. For downstream analyses, only cells for which clonotype information was available were retained. To remove likely doublet or multiplet captures, cells with more than 7,000 detected genes were discarded as well as cells for which more than two *TRA* or *TRB* sequences were detected. For SIFT-seq-based identification of (1) T cell clonotypes associated with a Mut *PIK3CA*-specific response, (2) gene expression pattern analyses of candidate clonotypes and (3) visualizations and TCR retrieval, custom R scripts were developed. V(D)J sequences of candidate T cell clonotypes were analyzed on the Loupe V(D)J browser 4.0.

### HLA-IP LC–MS/MS

COS-7 cells were co-electroporated with 100 μg ml^−1^ each of mRNA encoding an individual *HLA* allele with either full-length or truncated WT or Mut *PIK3CA* using the Neon Transfection system (100-μl tip, setting: 1,050 V/10 ms/2 pulses). Next, 15–20 × 10^6^ cells were electroporated per condition and plated in six-well non-TC plates overnight. Cells were harvested by incubating with 1 mM EDTA (Millipore Sigma) for 10 minutes at 37 °C. Harvested cells were pelleted and washed three times in ice-cold PBS (Gibco). Immunoprecipitation, HLA ligand separation and LC–MS/MS were performed as previously described^63^. In brief, cells were lysed in 7.5 ml of 1% CHAPS (Millipore Sigma) for 1 hour at 4 °C, lysates were spun down for 1 hour with 20,000*g* at 4 °C, and supernatant fluids were isolated. For immunopurification of HLA-I ligands, 0.5 mg of W6/32 antibody (Bio X Cell) was bound to 40 mg of CN Br-activated sepharose and incubated with the protein lysate overnight. HLA complexes and binding peptides were eluted five times using 1% TFA. Peptides and HLA-I complexes were separated using C18 columns (Sep-Pak C18 1 cc Vac Cartridge, 50 mg of sorbent per cartridge, 37–55-μm particle size, Waters). C18 columns were pre-conditioned with 80% ACN (Millipore Sigma) in 0.1% TFA and equilibrated with two washes of 0.1% TFA. Samples were loaded, washed again twice with 0.1% TFA and eluted in 300 μl of 30%, 40% and 50% acetonitrile in 0.1% TFA. All three fractions were pooled, dried down using vacuum centrifugation and stored at −80 °C until further processing. HLA-I ligands were isolated by solid-phase extractions using in-house C18 mini-columns. Samples were analyzed by high-resolution/high-accuracy LC–MS/MS (Lumos Fusion, Thermo Fisher Scientific). MS and MS/MS were operated at resolutions of 60,000 and 30,000, respectively. Only charge states 1, 2 and 3 were allowed. The isolation window was chosen as 1.6 Thomson, and collision energy was set at 30%. For MS/MS, maximum injection time was 100 ms with an automatic gain control of 50,000. MS data were processed using Byonic software (version 2.7.84, Protein Metrics) through a custom-built computer server equipped with four Intel Xeon E5-4620 8-core CPUs operating at 2.2 GHz and 512 GB physical memory (Exxact Corporation). Protein FDR was disabled to allow complete assessment of potential peptide identifications. Oxidization of methionine, phosphorylation of serine, threonine and tyrosine, as well as N-terminal acetylation were set as variable modifications for all samples. Samples were searched against a database comprising UniProt *Cercopithecus* *aethiops* reviewed proteins supplemented with human HLA allele sequences used in this study, WT and Mut sequences of PI3Kα, as well as common contaminants. For mirror plots, ion intensities were exported and re-plotted with GraphPad Prism. For precursor quantitation as well as retention time analyses, Skyline software (version 4.2, MacCoss Lab Software) was used. Precursor masses of peptide target sequences were searched in all relevant.raw files, and peak areas of all replicates were compared. Retention times for the best-scoring matches were selected with a total of four isotopes.

### TCR transduction

The 293GP packaging line was plated overnight onto poly-d-lysine-coated 60-mm^2^ plates at 1.6 × 10^6^ cells per plate in complete DMEM. 293GP cells were transfected with 6 μg of a candidate TCR pMSGV1-plasmid along with 3 μg of the RD114 envelope using the Lipofectamine 3000 (Invitrogen) reagent set. Both the pMSGV1 and RD114 plasmids were obtained through an MTA from S. A. Rosenberg. Viral supernatant was collected after 48 hours and loaded onto retronectin-coated (10 ug ml^−1^, Takara Bio) non-TC 24-well plates and centrifuged at 2,000*g* for 2 hours at 32 °C. CD8^+^ and CD4^+^ T cells enriched from HD polyclonal PBMCs were stimulated with plate-bound OKT3 (5 μg ml^−1^, Miltenyi Biotec), anti-CD28 (2 μg ml^−1^, Miltenyi Biotec) and rhIL-2 (300 IU ml^−1^) for 48 hours before transduction. Stimulated cells were plated at 1–5 × 10^5^ cells per well and spinoculated at 1,500 r.p.m. for 15 minutes. Cells were assessed for transduction efficiency after 3–4 days by measuring surface expression of mTCR by FACS.

### T cell immunoassays

Mutation specificity: Specificity of candidate TCRs was assessed by intracellular cytokine staining (ICS) using the BD Cytofix/CytoPerm Plus Kit, following the manufacturer’s instructions. Autologous moDCs from MSK 21LT2 and 0606T donors were electroporated with 100 μg ml^−1^ of mRNA encoding WT or Mut *PIK3CA* and plated into 96-well round-bottom plates overnight. To determine the class of HLA restriction, a set of plated moDCS were treated with an anti-HLA-A/B/C antibody (40 μg ml^−1^, Clone W6/32, BioLegend) or anti-class II (10 μg ml^−1^, Clone IVA12, MSKCC in-house) antibody for 3 hours at 37 °C. Candidate TCR-transduced T cells were co-cultured at an E:T ratio of 1:1 for 6 hours in the presence of anti-CD107A- BV650 (Clone H4A3, BioLegend) and Golgi block. PMA-ionomycin stimulation was included in all experiments as a positive control. Cells were washed in 1× PBS and surface labeled with Live/Dead fixable dye (Invitrogen), anti-CD3-APC-H7 (Clone SK7, Invitrogen), anti-CD4-Alexa Fluor 700 (Clone RPA-T4, Invitrogen), anti-CD8-efluor450 (Clone SK1, Invitrogen) and anti-mouse TCR-PerCpCy5.5 (Clone H57-597, Invitrogen) for 30 minutes at 4 °C. Cells were washed with 1× PBS and then fixed and permeabilized for 15 minutes at 4 °C. Surface-labeled cells were then washed with 1× perm-wash buffer and labeled with anti-IL-2-PE-Cy7 (Clone MQ1-17H12, Invitrogen), anti-TNFα-PE (Clone Mab11, Invitrogen) and anti-IFN-γ-FITC (Clone B27, BD) for 30 minutes at 4 °C in perm-wash buffer. All antibodies were used at a final concentration of 5 µg ml^−1^. Finally, cells were washed with perm-wash buffer, suspended in 2% FBS in PBS and acquired on an X20 LSR Fortessa flow cytometer with the BD FACSDiva software. Data were analyzed using FlowJo software version 10.6.2. Representative gating strategies are shown in Supplementary Figs. 12–15. HLA-restriction determination: COS-7 cells were co-electroporated with 100 μg ml^−1^ each of mRNA encoding an individual *HLA* allele and either WT or Mut *PIK3CA*. Transfected COS-7 cells were plated overnight in a 96-well round-bottom plate in complete media to allow surface p/HLA-I complex expression. TCR-transduced T cells were added in at an E:T ratio of 1:1, and cytokine production was measured by ICS as described above. TCR coreceptor dependence and functional avidity: Target cells expressing HLA-A*03:01 were electroporated with titrated quantities of mRNA encoding Mut or WT *PIK3CA* and plated in 96-well round-bottom plates overnight. Enriched CD4^+^ or CD8^+^ T cells expressing an individual Mut *PIK3CA*-specific TCR were added in at a 1:1 E:T ratio. EC_50_ values for individual TCRs were calculated by determining the antigen concentration that elicits 50% of maximal response (EC_50_). TCR killing assay: TCR cytolytic capability was measured by a tumor impedance assay using the xCELLigence System (ACEA Biosciences). Target cells expressing HLA-A*03:01 and either Mut or WT *PIK3CA* were plated overnight into custom xCELLigence E-plate 96-well flat-bottom plates. Before adding T cells, a baseline impedance measurement was taken. TCR-transduced T cells were added at an E:T ratio of 1:4, and impedance measurements were recorded at 15-minute intervals for up to 96 hours. Targets plated in complete media or exposed to 1% Triton X-100 allowed minimum and maximum lysis measurements, respectively. Percent cytolysis was calculated using RTCA Software Pro. TCR recognition motif: Target cells expressing HLA-A*03:01 were pulsed with pWT, pMut or variants of pMut that contain an Ala or Gly substitution at each individual peptide position (1 μg ml^−1^) for 60 minutes at 37 °C. Unbound peptide was washed off, and either TCR3-transduced or TCR4-transduced T cells were added at an E:T ratio of 1:1. Each TCR’s recognition motif was defined by positions resulting in ≥50% loss of function with an AA substitution compared to the native peptide sequence as previously described^64^. TCR4 cross-reactivity screening: ScanProsite was used to search all UniProtKB/Swiss-Prot database sequences, including splice variants, for proteins containing the motif ‘X-X-X-X-G-W-T-T-K’. No filters were used. PDX reactivity: An established PDX (USC_X10) derived from an HLA-A*03:01^+^ patient with *PIK3CA* (H1047L)^+^ uterine serous cancer was explanted from an immune-deficient NSG mouse. Tumor cells were digested by enzymatic disruption with DNase I and collagenase IV, followed by filtering through a 100-µm filter to obtain a single-cell digest. Tumor cells were labeled with Live/Dead Fixable Dye (Invitrogen), anti-HLA-A/B/C-PerCpCy5.5 (Clone W6/32, BioLegend) and anti-HLA-A*03 (Clone GAP.A3, Invitrogen) for 30 minutes at 4 °C. Cells were washed with 1× PBS and resuspended in 2% FBS in PBS before acquisition by FACS. Then, 1 × 10^5^ tumor cells were co-cultured with TCR4-tranduced T cells at an E:T ratio of 1:1 overnight at 37 °C in the absence or presence of an anti-HLA-A/B/C blocking antibody (40 μg ml^−1^, Clone W6/32). TCR4-transduced T cells cultured alone were used as a negative control. Reactivity was measured by changes in 4-1BB expression on mTCR^+^CD8^+^ T cells using anti-CD137-APC (Clone 4B4-1, BD Biosciences).

### ACT xenograft model

All animal procedures were performed in accordance with an MSKCC Institutional Animal Care and Use Committee-approved protocol (19-08-013). Four to six-week-old female NOD.Cg-*Prkdc*^scid^ *Il2rg*^*tm1Wjl*^/SzJ (NSG) mice were purchased from Jackson Laboratory and housed in pathogen-free conditions at the MSKCC vivarium. The mouse room maintained a 12-hour light/dark cycle, temperature of 65–75 ^°^F and humidity levels of 40–60%. Three × 10^6^ cells of the indicated HCC70 isogenic cell lines (HCC70-WT *PIK3CA* or HCC70-Mut *PIK3CA*) were subcutaneously implanted into the right flank of mice. Mice were randomized to indicated treatment groups once tumors were established to ~75 mm^3^. Tumor width and diameter as well as animal weights were measured by an investigator blinded to treatment conditions at baseline and twice weekly thereafter. TCR4-tranduced CD8^+^ T cells were intravenously transferred at day 3 (7.5 × 10^6^) and day 7 (2.5 × 10^6^). Control groups received an equal number of CD8^+^ T cells transduced with a previously described HLA-A*03:01-restricted Flu-specific TCR^65^ or PBS. Mice simultaneously received IL-15/IL-15Rα complex intraperitoneally every 3 days for the duration of the experiment, starting at the time of the first T cell transfer. The complex was freshly prepared by incubating recombinant human IL-15 (Miltenyi Biotec) with IL-15R-α-Fc (R&D Systems) at a 1:1 molar ratio for 30 minutes at 37 °C and administered at the final concentration of 1 μg of IL-15 per mouse. The maximum tumor size permitted by the approved protocol is 1,000 mm^3^ and this value was not exceeded.

### Dextramer labeling

HLA-A*03:01 multimers bound to pMut and conjugated to PE or APC were purchased from Immudex. Cells were labeled with dual fluorophore-conjugated dextramers for 10 minutes at room temperature, followed by surface antibodies against CD3, CD4, CD8 and viability dye for an additional 20 minutes at 4 °C. Cells were washed and acquired on a BD Fortessa X20 flow cytometer.

### Recombinant protein synthesis and isolation

Proteins for biophysical assays and crystallography, including the HLA-A*03:01 heavy chain, β_2_-microglobulin (β_2_m), the S3-4 pan-HLA TCR binding variant and the TCR α/β chains, were expressed in *Escherichia coli* as inclusion bodies and dissolved in 8 M urea and 6 M guanidinium-HCl. Denatured proteins were refolded and purified in vitro as previously described^66,67^. In brief, for TCR folding, TCR α/β chains at a 1:1 ratio were diluted into TCR refolding buffer (50 mM Tris-HCl, 2.5 M urea, 2 mM NaEDTA, 6.5 mM cysteamine, 3.7 mM cystamine and 0.2 mM PMSF, pH 8.15) and incubated at 4 °C overnight. The refolding buffer was then dialyzed against ddH_2_O and 10 mM Tris-HCl (pH 8.3) at 4 °C for 36 hours. For p/HLA folding, human β_2_m and HLA-A*03:01 were injected into the refolding buffer (400 mM L-arginine, 100 mM Tris-HCl, 2 mM NaEDTA, 6.3 mM cysteamine, 3.7 mM cystamine and 0.2 mM PMSF, pH 8.30), respectively, at a 3:1 ratio in the presence of a ten-fold excess of peptides at 4 °C. After an overnight incubation, the solution was dialyzed against ddH_2_O and 10 mM Tris-HCl (pH 8.3) at room temperature for 48 hours. The refolded proteins were then purified via ion exchange, followed by size-exclusion chromatography. Protein concentrations were determined through UV absorbance using sequence-determined extinction coefficients. WT and Mut PI3Kα peptides were purchased from AAPPTec or GenScript at >80% purity, diluted to 30 mM in DMSO and stored at −80 °C.

### Differential scanning fluorimetry

Melting temperatures of WT and Mut PIK3α peptide/HLA-A*03:01 complexes were measured via differential scanning fluorimetry on a StepOnePlus Real-Time PCR System (Applied Biosystems), as previously described^68^. In brief, a 96-well plate was loaded with mixtures of 2 μl of 100× SYPRO Orange Protein Gel Stain (Invitrogen) and 18 μl of target protein at 15 μM in HBS-EP buffer (10 mM HEPES, 150 mM NaCl, 3 mM NaEDTA, 0.005% surfactant P20, pH 7.4). The excitation and emission wavelengths for the measurement were 587 nm and 607 nm, respectively. Temperature was scanned from 20 °C to 95 °C at a rate of 1 °C min^−1^. Melting temperatures were determined by fitting the 1st derivative of each melting curve to a bi-Gaussian function in OriginPro.

### Fluorescence anisotropy

Dissociation of WT and Mut PI3Kα peptides from peptide/HLA-A*03:01 complexes was determined through fluorescence anisotropy, as previously described^69^. In brief, purified peptide/HLA-A:03:01 complexes were generated using derivatives of the pWT and pMut peptides where the P5 Gly was substituted with a 5-carboxyfluorescein-modified lysine. Experiments were performed on a Beacon 2000 Fluorescence Polarization instrument (Invitrogen). Next, 100 nM fluorescein-labeled peptide/MHC complexes were mixed with 100 μM unlabeled peptide in 20 mM NaH_2_PO_4_ and 75 mM NaCl (pH 7.4). The excitation wavelength was 488 nm, and anisotropy was detected at 535 nm. Changes in anisotropy were recorded as a function of time. Dissociation kinetics of peptides were determined by fitting the anisotropy curve to a single or biphasic exponential decay function in MATLAB. Half-lives were computed from the slowest dissociation rate from the relationship t_1/2_ = 0.693/k_off_.

### Protein crystallization

Purified proteins were exchanged into 10 mM HEPES and 20 mM NaCl (pH 7.4) before crystallization. Crystals of pWT/HLA-A*03:01 grew in 10% PEG 8000, 200 mM calcium acetate and 100 mM HEPES (pH 7.5) at a protein concentration of 15 mg ml^−1^. Crystals of pMut/HLA-A*03:01 grew in 20% PEG 3350 and 200 mM ammonium formate (pH 6.6) at a protein concentration of 15 mg ml^−1^. Crystals of the TCR3-pMut/HLA-A*03:01 and TCR4-pMut/HLA-A*03:01 complexes grew in 10% PEG 8000 and 200 mM magnesium acetate at a protein concentration of 5 mg ml^−1^. Crystals were obtained by hanging drop vapor diffusion at 4 °C and were cryoprotected with 15% glycerol and flash-frozen before data collection.

### X-ray diffraction and structure determination

X-ray diffraction was performed at the beamline 22-ID or 24-ID-E of the Advanced Photon Source at Argonne National Laboratory. Diffraction data were processed with HKL2000 and solved by molecular replacement via Phaser in Phenix. The search model for the HLA-A*03:01 complexes was PDB 2XPG^70^. The search models for the complexes with TCR3 and TCR4 were built by Sculptor with the α chain from Protein Data Bank (PDB) 3QH3 and the β chain from PDB 4PRH^71,72^. The peptide and CDR loops were removed from the models before molecular replacement and manually rebuilt in Coot after the model was obtained from Phenix AutoBuild. Models were further refined automatically in Phenix and manually in Coot. Structures were visualized using PyMOL 2.3.4 and Discovery Studio 2019.

### SPR

Binding measurements between either TCR3 or TCR4 and the pMut/HLA-A*03:01 and pWT/HLA-A*03:01 complexes were performed using SPR on a Biacore T200 instrument, as previously described^37,67^. In brief, proteins were exchanged into 10 mM HEPES, 150 mM NaCl, 3 mM EDTA and 0.005% surfactant P-20, pH 7.4. The TCRs were immobilized on a CM5 Series S sensor chip to 900−1,300 response units via amine coupling. Experiments were performed at 25 °C with blank activated and deactivated flow cells as a reference. pMHC complexes were injected at a flow rate of 5 μl ml^−1^ as analytes in duplicate. A pan HLA-I TCR variant that binds the ‘side’ of HLA-I molecules was used to verify the stability of the p/HLA complex during SPR measurements^67^. Binding affinities were determined by fitting the steady-state data from both sets of injections to a 1:1 binding model in OriginPro.

### *PIK3CA* clonality, tumor phylogeny and *HLA* LOH

The proportion of cancer cells harboring *PIK3CA* (H1047L) (CCF) was estimated by integrating the number of copies of the mutant allele, the purity of the tumor and the variant allele frequency of the mutation^73^. Allele-specific copy number inferences were made using FACETS^74^, and the number of copies of the mutant allele was inferred as described previously^75^. Mutations were deemed clonal if the CCF value is >0.8 or if the CCF value is >0.7 and the upper bound of the 95th percentile confidence interval is >0.9. All mutations for which CCF was determinable but that did not meet the criteria for being clonal were designated as sub-clonal. HLA genotypes from MSK-IMPACT were inferred using POLYSOLVER^44,76^. LOH at HLA-I alleles was inferred using LOHHLA^48^. At each of the HLA-I loci, LOH was called if the median copy number calculated by LOHHLA was less than 0.5 and the *P* value reflecting the allelic imbalance was less than 0.001. To visualize LOH at the *HLA* gene locus, we identified mismatched sites between the two *HLA-A* alleles and then computed the total number of reads aligning to each mismatched site in each tissue site.

### Statistics and reproducibility

No statistical methods were used to predetermine sample size. Appropriate statistical tests were used to analyze data, as described in each figure legend. Statistical analyses were performed with GraphPad Prism version 8.4 software. Significance was preset at *P* < 0.05. In vitro experimental data were generated from two or more independent experiments containing *n* = 3 biological replicates per condition per experiment. For in vivo mouse experiments, treatment groups had *n* = 10 mice per condition, and control groups had *n* = 5 mice. Mice were randomized into three groups after tumor implantation. An investigator blinded to treatment groups performed both data acquisition and data analysis. In vivo experiments were independently repeated two times, and all attempts at replication were successful. For both in vitro and in vivo experiments, no data were excluded from any analysis.

### Reporting Summary

Further information on research design is available in the Nature Research Reporting Summary linked to this article.

## Online content

Any methods, additional references, Nature Research reporting summaries, source data, extended data, supplementary information, acknowledgements, peer review information; details of author contributions and competing interests; and statements of data and code availability are available at 10.1038/s41591-022-01786-3.

## Supplementary information

Supplementary Information Supplementary Figs. 1–15 and Supplementary Tables 1–5

Reporting Summary

## Data Availability

Single-cell RNA sequencing datasets are deposited in the Gene Expression Omnibus database (GSE172403). Structural data, including coordinates and structure factors of pWT/HLA-I, pMut/HLA-I and the ternary TCR4/pMut/HLA-I and TCR3/pMut/HLA-I complexes, are available at the Protein Data Bank (https://www.rcsb.org/) under PDB accession codes 7L1B, 7L1C, 7L1D and 7RRG. Additional materials are available upon reasonable request to C.A.K. (klebanoc@mskcc.org).

## Code availability

Custom codes to analyze SIFT-seq data are available on GitHub at https://github.com/abcwcm/Chandran2021.
