# Supplementary material for: Immunogenicity and therapeutic targeting of a public neoantigen derived from mutated PIK3CA
Source: Nat Med. 2022 Apr 28;28(5):946–57. doi: 10.1038/s41591-022-01786-3 (PMC9117146; doi:10.1038/s41591-022-01786-3)
Supplement: Supplementary file 1 — Supplementary Figs. 1–15 and Supplementary Tables 1–5 [file 41591_2022_1786_MOESM1_ESM.pdf]

nature portfolio

https://doi.org/10.1038/s41591-022-01786-3

Supplementary information

Immunogenicity and therapeutic targeting of a public neoantigen derived from mutated *PIK3CA*

In the format provided by the authors and unedited

Supplementary Fig. 1: Negative and positive predictive capacity of the SIFT-seq discovery platform for Mut *PIK3CA*-specific TCRs.

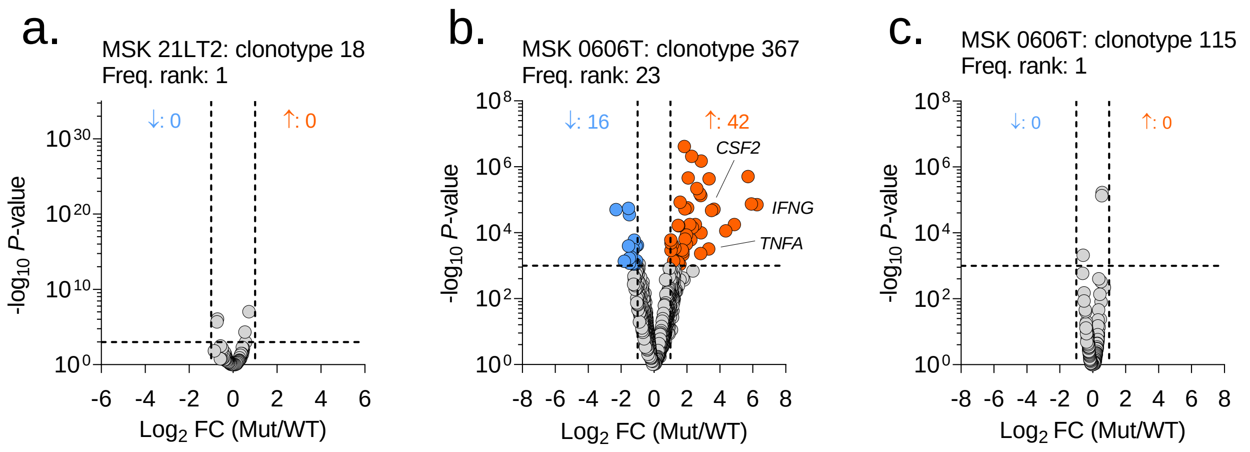

Volcano plots displaying global transcriptomic changes for (**a**) MSK 21LT2 clonotype 18 (non-reactive; frequency rank = 1), (**b**) MSK 0606T clonotype 367 (reactive; frequency rank = 23), and (**c**) MSK 0606T clonotype 115 (non-reactive; frequency rank = 1) following stimulation with Mut versus WT *PIK3CA*. Vertical and horizontal dashed lines indicate thresholds for gene expression $\log_2$ fold-change (FC) and statistical significance, respectively. Orange and blue dots represent significantly up and down-regulated genes following Mut *PIK3CA* stimulation, respectively.

Supplementary Fig. 2: T cells transduced with TCRs retrieved from MSK 21LT2 clonotype 20 or MSK 0606T clonotype 367 generate a polyfunctional Mut *PIK3CA*-specific cytokine response.

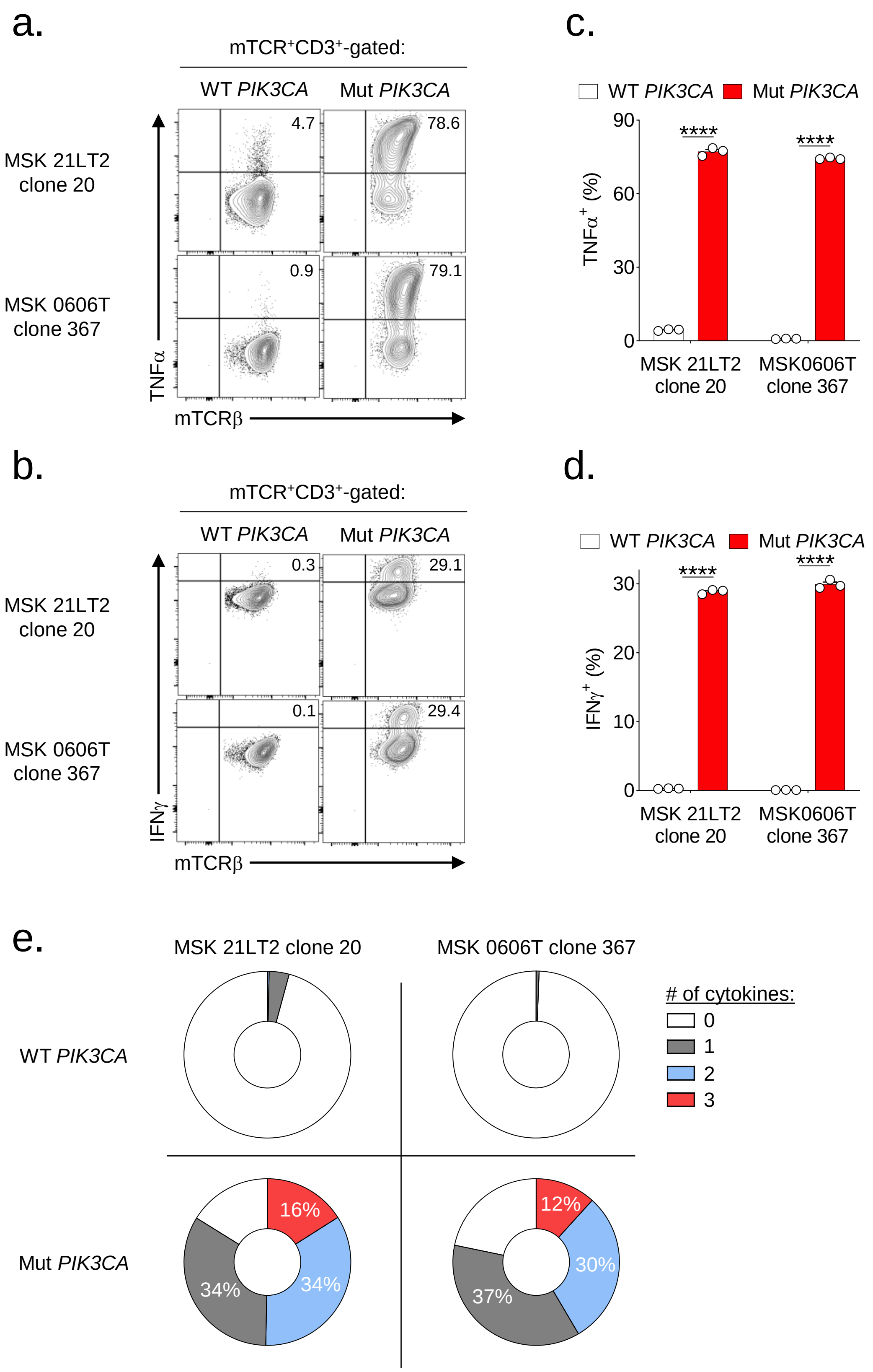

Supplementary Fig. 2, continued:
(**a, b**) Representative FACS plots, (**c, d**) summary bar graphs, and (**e**) polyfunctionality circus plot illustrating TNFα, IFNγ, and IL-2 production in T cells retrovirally transduced with SIFT-seq retrieved TCR gene sequences. A polyclonal population of T cells was transduced with either the TCR retrieved from MSK 21LT2 clonotype 20 or MSK 0606T clonotype 367. Transduced T cells were co-cultured with autologous monocyte derived DCs electroporated with mRNA encoding Mut or WT *PIK3CA*. Bar graphs displayed as mean ± SEM using n=3 biologic replicates per condition. ****P < 0.0001 using a two-sided Student's t-test.

Supplementary Fig. 3: Normalization of the *IFNG* signal within candidate clonotypes under Mut versus WT stimulation conditions enhances the specificity of identifying mutation-reactive TCRs from MSK 21LT2.

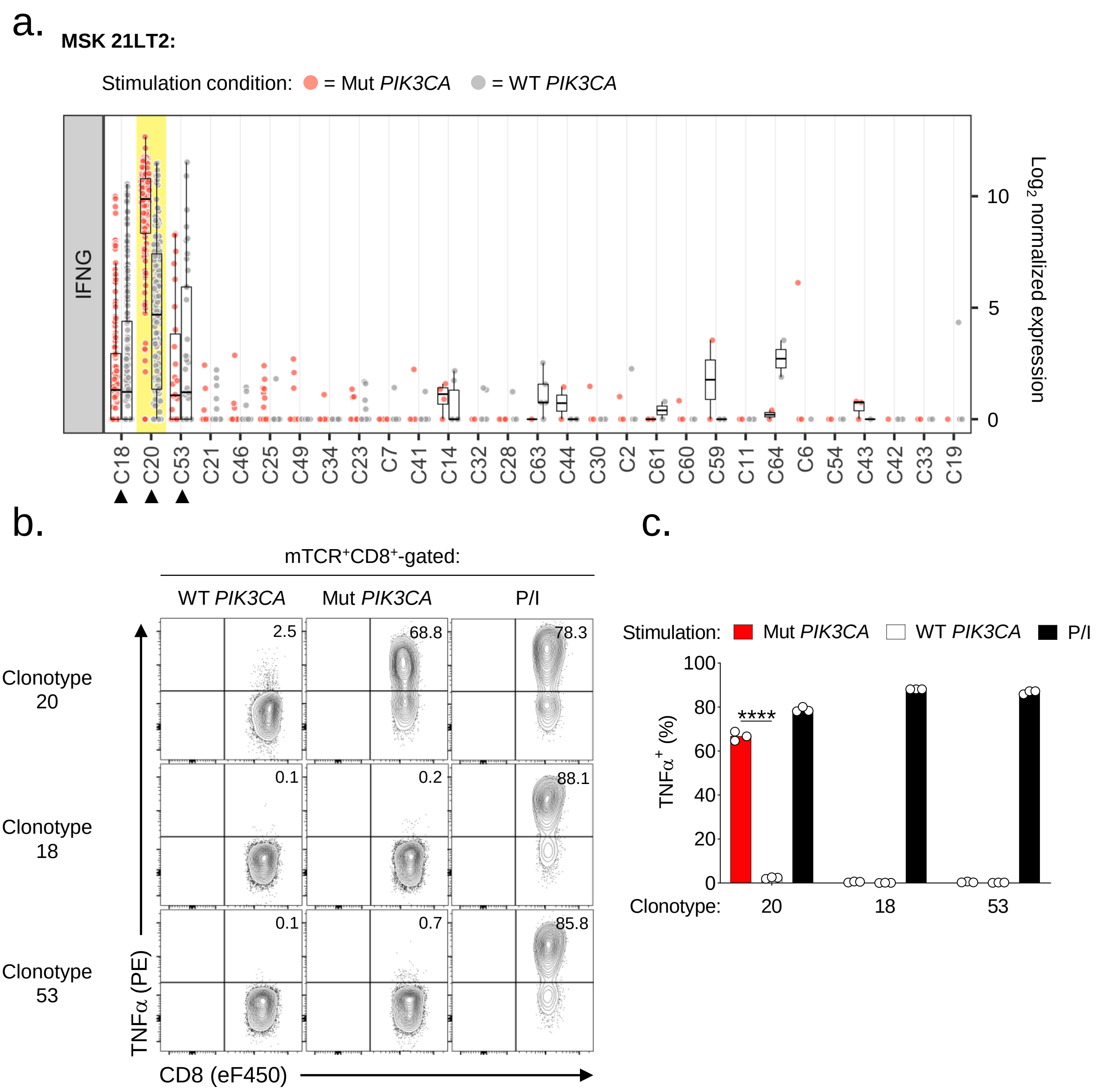

Supplementary Fig. 3, continued:

(**a**) Absolute values for *IFNG* transcripts from *n*=29 unique TCR clonotypes identified using single-cell RNA and V(D)J TCR sequencing from screen positive "hit" well MSK 21LT2. Matched aliquots of sensitized T cells from healthy donor (HD) 1 were stimulated with *PIK3CA* (H1047L) (Mut) or wildtype *PIK3CA* (WT) prior to single-cell sequencing. The median (solid black line), upper quartile (top of box), lower quartile (bottom of box), and range (whiskers) for *IFNG* values from all evaluable TCR clonotypes under each stimulation condition is shown. Values from the SIFT-seq retrieved Mut *PIK3CA*-specific TCR clonotype 20 is highlighted in yellow; (▲) indicates clonotypes selected for TCR reconstruction and functional testing. (**b**) Representative FACS plots and (**c**) summary bar graphs (*n*=3 biologic replicates per condition) displaying the frequency of intra-cellular TNFα production in polyclonal T cells retrovirally transduced with TCR clonotypes 18, 20, and 53. All reconstructed TCRs express a murine constant chain (mTCR), enabling detection with an anti-mTCR-specific antibody. Transduced $CD8^+$ T cells ($live^+mTCR^+CD8^+$) were co-cultured with target cells co-expressing HLA-A*03:01 and either WT or Mut *PIK3CA*. Stimulation by phorbol 12-myristate 13-acetate-ionomycin (P/I) was used as a TCR-independent positive control for T cell function. Bar graphs displayed as mean ± SEM. ****$P < 0.0001$ using a two-sided Student's t-test.

Supplementary Fig. 4: Normalization of the *IFNG* signal within candidate clonotypes under Mut versus WT stimulation conditions enhances the specificity of identifying mutation-reactive TCRs from MSK 0606T.

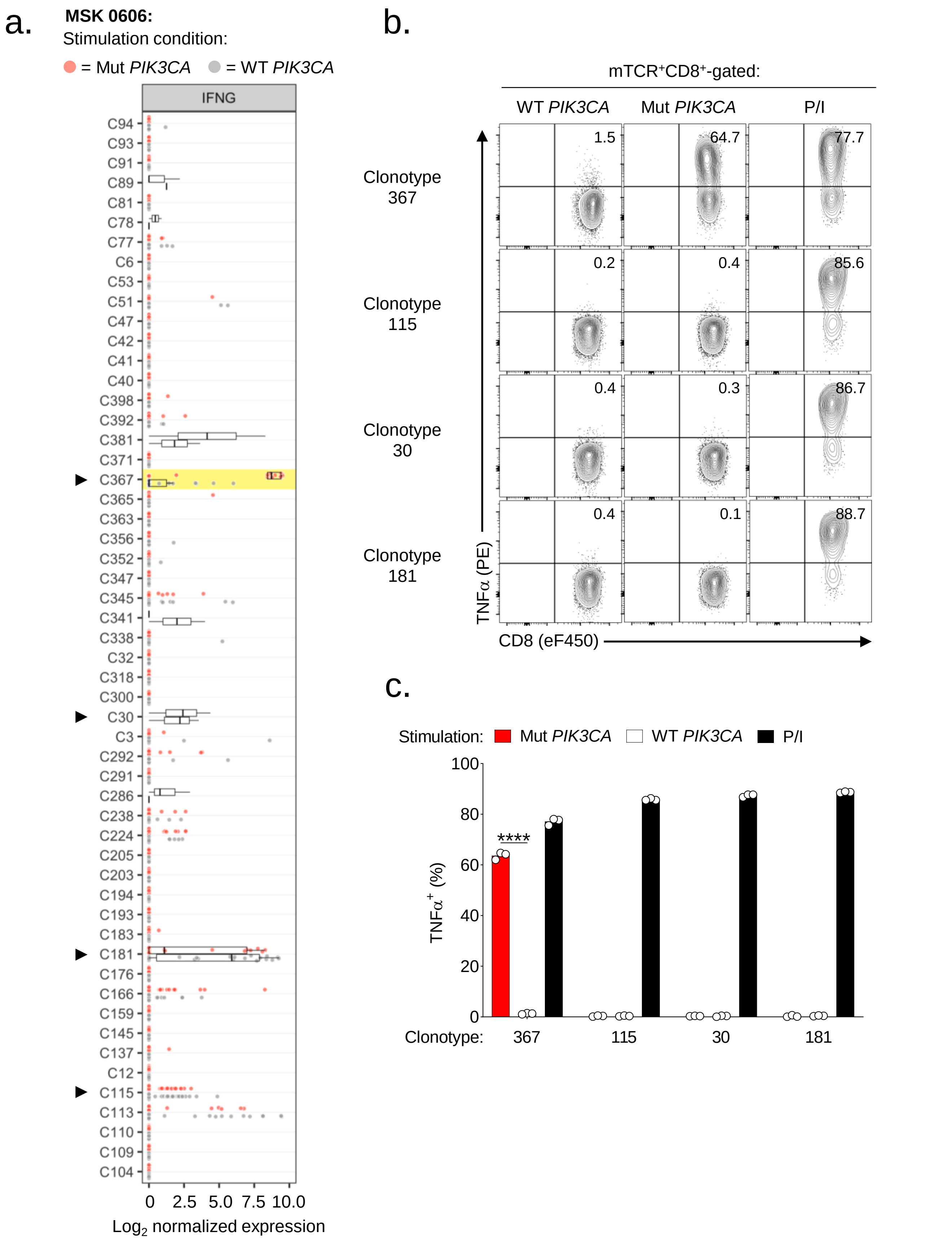

Supplementary Fig. 4, continued:
(**a**) Absolute values for *IFNG* transcripts from *n*=54 unique TCR clonotypes identified using single-cell RNA and V(D)J TCR sequencing from screen positive "hit" well MSK 0606T. Matched aliquots of sensitized T cells from healthy donor (HD) 2 were stimulated with *PIK3CA* (H1047L) (Mut) or wildtype *PIK3CA* (WT) prior to single-cell sequencing. The median (solid black line), upper quartile (top of box), lower quartile (bottom of box), and range (whiskers) for *IFNG* values from all evaluable TCR clonotypes under each stimulation condition is shown. Values from the SIFT-seq retrieved Mut *PIK3CA*-specific TCR clonotype 367 is highlighted in yellow; (▲) indicates clonotypes selected for TCR reconstruction and functional testing. (**b**) Representative FACS plots and (**c**) summary bar graphs (*n*=3 biologic replicates per condition) displaying the frequency of intra-cellular TNFα production in polyclonal T cells retrovirally transduced with TCR clonotypes 30, 115, 181, and 367. All reconstructed TCRs express a murine constant chain (mTCR), enabling detection with an anti-mTCR-specific antibody. Transduced $CD8^+$ T cells ($live^+mTCR^+CD8^+$) were co-cultured with target cells co-expressing HLA-A*03:01 and either WT or Mut *PIK3CA*. Stimulation by phorbol 12-myristate 13-acetate-ionomycin (P/I) was used as a TCR-independent positive control for T cell function. Bar graphs displayed as mean ± SEM. ****$P < 0.0001$ using a two-sided Student's t-test.

Supplementary Fig. 5: Comparison of mutation-specific effector functions for a panel of *PIK3CA* public neoantigen TCRs.

a.

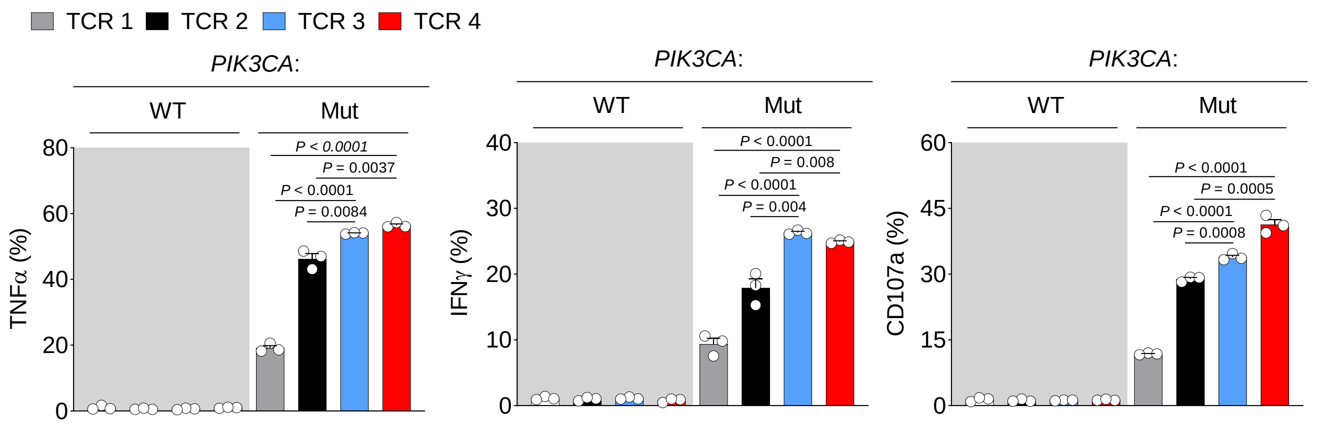

b.

TCR ID	EC_{50} $CD8^+$ (μM)	EC_{50} $CD4^+$ (μM)
TCR1	169	-
TCR2	0.268	-
TCR3	0.029	0.489
TCR4	0.026	0.769

(**a**) Summary bar graphs demonstrating Mut-specific expression of multiple effector molecules by T cells transduced with a *PIK3CA* public NeoAg TCR. Polyclonal $CD8^+$ T cells were individually transduced with *PIK3CA* public NeoAg TCR panel members 1-4 and cocultured with HLA-A*03:01$^+$ target cells that express either WT or Mut *PIK3CA*. Numbers in each bar graph indicate the frequency of T cells producing TNFα, IFNγ, or CD107a measured by FACS analysis after pre-gating on live$^+$mTCR$^+$CD8$^+$ cells. Bar graphs are displayed as mean ± SEM using *n*=3 biologic replicates per condition. Exact *P* values are displayed above each comparison and were calculated using a two-sided Student's t-test. (**b**) Mean effective concentration of Mut *PIK3CA* required to trigger half-maximal (EC_{50}) TNFα production in enriched $CD8^+$ or $CD4^+$ T cells transduced with a *PIK3CA* public NeoAg TCR. $CD8^+$ or $CD4^+$ T cells were individually transduced with PIK3CA public NeoAg TCRs 1-4. Transduced T cells were co-cultured with an HLA-I mono-allelic cell line expressing HLA-A*03:01 and electroporated with defined concentrations of Mut *PIK3CA* mRNA. All data shown is representative of *n*=2 independent experiments.

Supplementary Fig. 6: An immuno-peptidomic screen for endogenously processed and HLA-A*03:01 presented peptides derived from wildtype *PIK3CA*.

a.

ID	PI3Kα amino acid sequences	NetMHC predicted 9mer peptides	Mass-spec identified 9mer peptides
Full-length *PIK3CA* (H1047L)	1-1068	16	2
WT *PIK3CA* minigene 1	1-373	8	3
WT *PIK3CA* minigene 2	349-721	7	0
WT *PIK3CA* minigene 3A	697-1068	0	0
PIK3CA (H1047L) minigene 3B	697-1068	1	1

b.

Mass-spec detected (+/-):

	NetMHC predicted epitope	E.L. % rank	B.A. (nM)	Mini-gene	Full-length
1.	ITIKHELFK	0.11	27.4	-	-
2.	KVIEPVGNR	0.09	174.7	+	-
3.	QVIAEAIRK	0.41	513.4	-	-
4.	VIAEAIRKK	0.08	115.1	+	+
5.	MLLSSEQLK	0.33	66.0	-	-
6.	YMNGETSTK	0.38	197.6	+	-
7.	VINSALRIK	0.16	75.2	-	-
8.	KIYVRTGIY	0.31	29.3	-	-
9.	GVTGSNPNK	0.38	469.7	-	-
10.	VTIPEILPK	0.05	79.3	-	-
11.	LVKDWPPIK	0.28	375.0	-	-
12.	NLLVRFLLK	0.44	44.3	-	-
13	LLVRFLLKK	0.22	34.4	-	-
14.	HLKSEMHNK	0.13	105.3	-	-
15.	GMYLKHLNR	0.29	85.5	-	-
16.	ALHGGWTTK	0.03	35.3	+	+
Wild-type control	AHHGGWTTK	4.012	9840.7	-	-

Relative value

Low High

Supplementary Fig. 6, continued:
(**a**) Table listing the full-length and minigene *PIK3CA* constructs tested, the total number of high-affinity nine amino acid peptides (9mers) predicted by NetMHCpan 4.1 to bind to HLA-A*03:01, and the number of HLA immunoprecipitation/liquid chromatography tandem mass-spectrometry (HLA-IP/MS) detected peptides. Strong HLA binding was defined as an eluted ligand (E.L.) % rank of <0.5% and/or peptide binding affinity (B.A.) <500 nM. (**b**) Summary table listing the identity of 9mer peptide sequences predicted to bind with high-affinity to HLA-A*03:01, the peptide's corresponding NetMHCpan 4.1 predicted E.L. % rank and B.A., and whether the peptide was detected by HLA-IP/MS. Heat map color indicates either the relative value for the NetMHCpan 4.1 prediction (red = low, blue = high) or whether a peptide sequence was detected by HLA-IP/MS (red = yes, blue = no).

Supplementary Fig. 7: Structural details and comparisons of the PI3Kα pMut and pWT peptides bound to HLA-A*03:01.

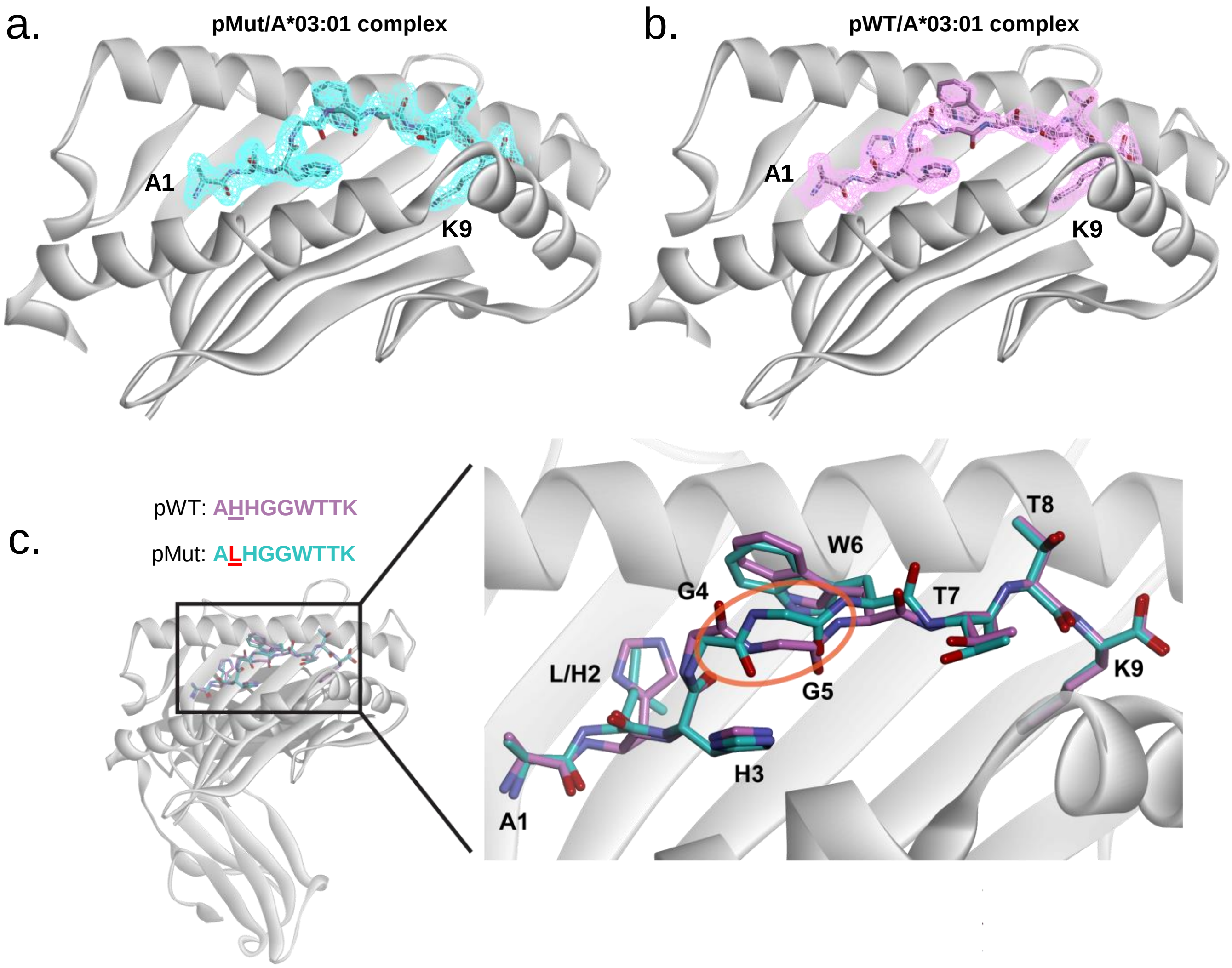

Electron density of the (**a**) pMut and (**b**) pWT peptides in the HLA-A:03:01 binding groove with $2F_o$-F_c contoured at 1σ. (**c**) Comparison of the peptide conformations in the binding grooves. A small structural divergence is present at the backbone at P4 and P5 Gly residues but does not alter overall peptide conformation or amino acid side chains positions.

Supplementary Fig. 8: Measurement of the thermal stability of a *PIK3CA* public neopeptide or its wildtype counterpart bound to HLA-A*03:02 using differential scanning fluorimetry.

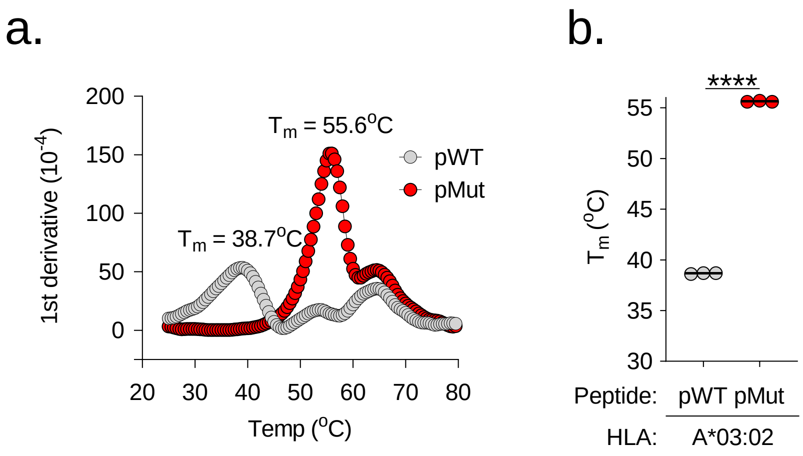

(**a**) Representative thermal melt curves and (**b**) summary scatter plot displaying the melting temperatures (T_m) of the pMut and wild type peptide (pWT) / HLA-A*03:02 complexes using differential scanning fluorimetry. Symbols are displayed as mean ± SEM using n=3 technical replicates. ****$P \leq 0.0001$ using a two-sided Student's t-test.

Supplementary Fig. 9: Comparison of the *in vitro* function of CD8+ T cells transduced with TCR4 or an HLA-A*03:01-restricted influenza control TCR.

a.

Antigen class	Antigen	Restricting HLA allele	Minimal epitope	Peptide length	B.A. (nM)
Public neoantigen	PI3Kα (H1047L)	A*03:01	A**L**HGGWTT**K**	9	35.3
Self antigen	PI3Kα	A*03:01	AHHGGWTT**K**	9	9840.7
Viral antigen	Influenza NP	A*03:01	I**L**RGSVAH**K**	9	28.4

b.

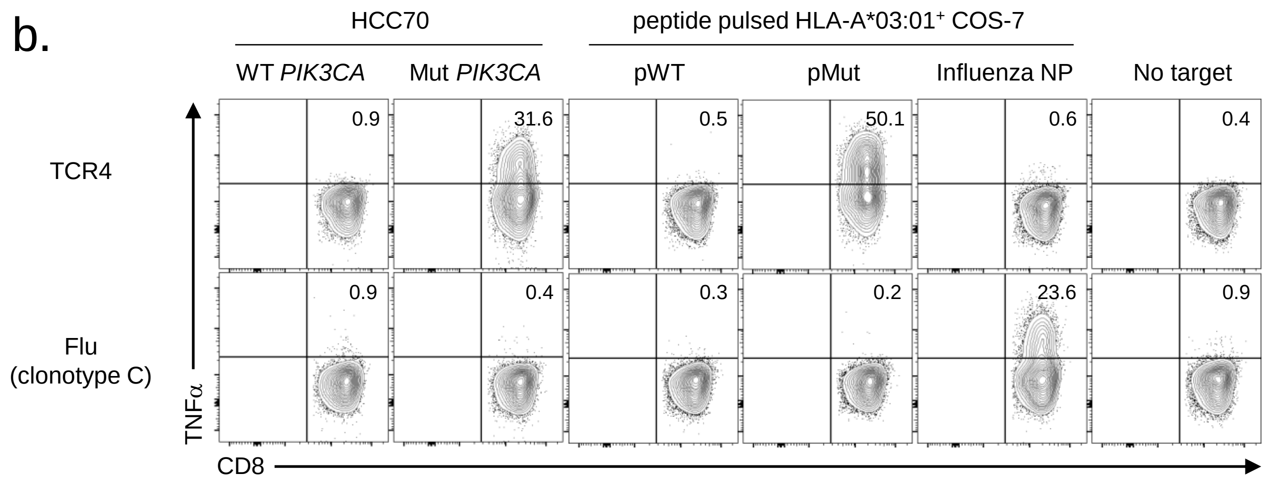

c.

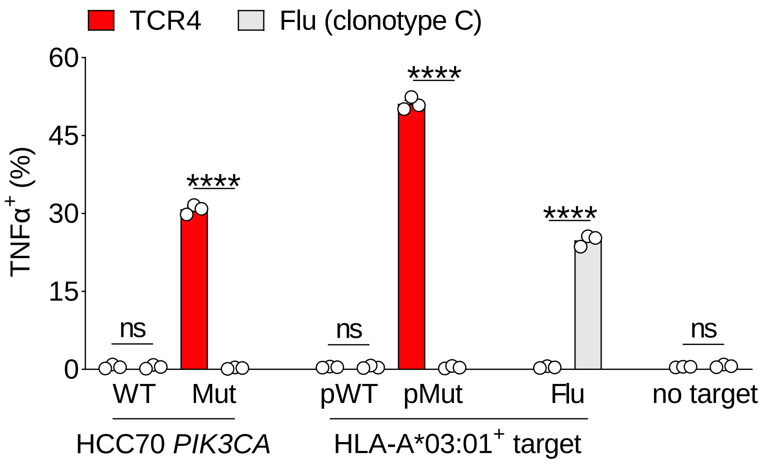

Supplementary Fig. 9, continued:
(**a**) Table comparing the biophysical features of the PI3Kα pMut, PI3Kα pWT, or an influenza nucleoprotein (Flu) control peptide. Predicted binding affinity (B.A.) based on NetMHCpan 4.1. **X** = preferred HLA-I anchor residue. (**b**) Representative FACS plots and (**c**) summary bar graph of intracellular TNFα production by $CD8^+$ T cells transduced with TCR4 or a Flu-specific control TCR (clonotype C). T cells were cocultured with HCC70-WT *PIK3CA*, HCC70-Mut *PIK3CA*, or HLA-A*03:01$^+$ COS-7 cells pulsed with 1μM of the indicated peptides. Numbers within each plot indicate the frequency of TNFα producing TCR transduced $CD8^+$ T cells after pre-gating on live$^+$mTCR$^+$ T cells. Results shown as mean ± SEM using *n*=3 biologic replicates per condition. ****$P < 0.0001$ and ns = not significant using a two-sided Student's t-test with Bonferroni correction.

Supplementary Fig. 10: Individual tumor growth curves for mice receiving adoptively transferred T cells genetically engineered with a *PIK3CA* public NeoAg-specific TCR, Flu-specific TCR, or phospho-buffered saline.

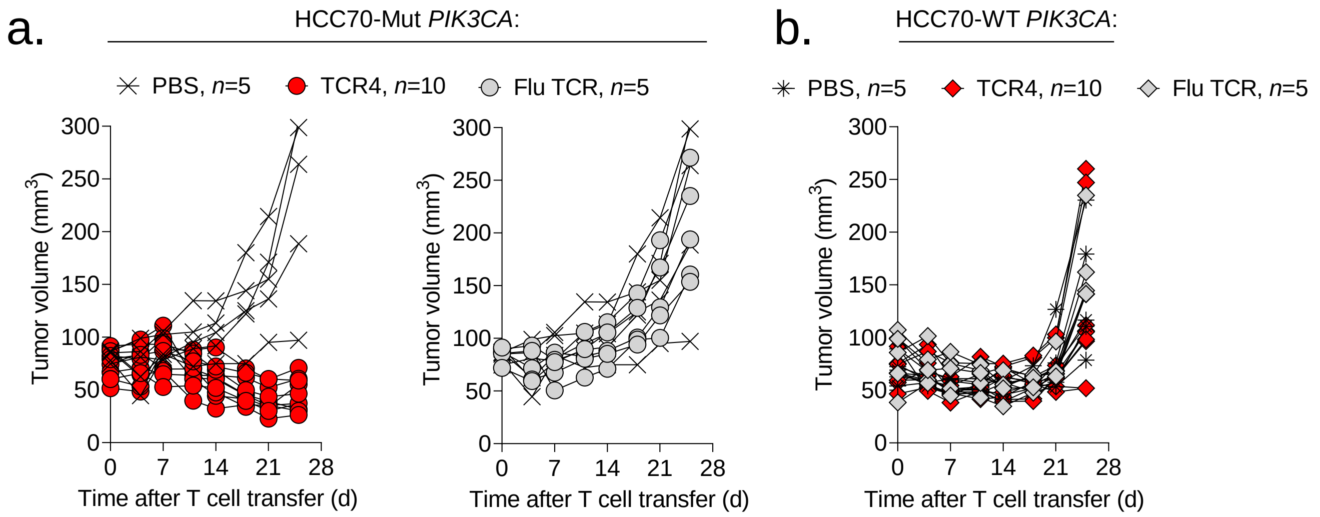

Tumor growth kinetics from individual mice in the experiment displayed in **Fig. 5b** and **Fig. 5d**. Mice implanted with either (**a**) HCC70-Mut *PIK3CA* tumors or (**b**) HCC70-WT *PIK3CA* tumors received twice weekly intraperitoneal injections of 1μg of IL-15 pre-complexed with IL-15Rα–Fc (1:1M) in addition to intravenous injection of either $CD8^+$ T cells transduced with TCR4, $CD8^+$ T cells transduced with an influenza (Flu)-specific TCR, or phosphate-buffered saline (PBS). $7.5e^6$ and $2.5e^6$ TCR^+ T cells were administered on D0 and D3 after randomization. TCR4 *n*=10, Flu TCR *n*=5, PBS *n*=5.

Supplementary Fig. 11: Adoptive transfer of TCR4-transduced T cells does not cause significant changes in the weights of mice bearing established Mut or WT *PIK3CA expressing* tumors compared with control treated mice.

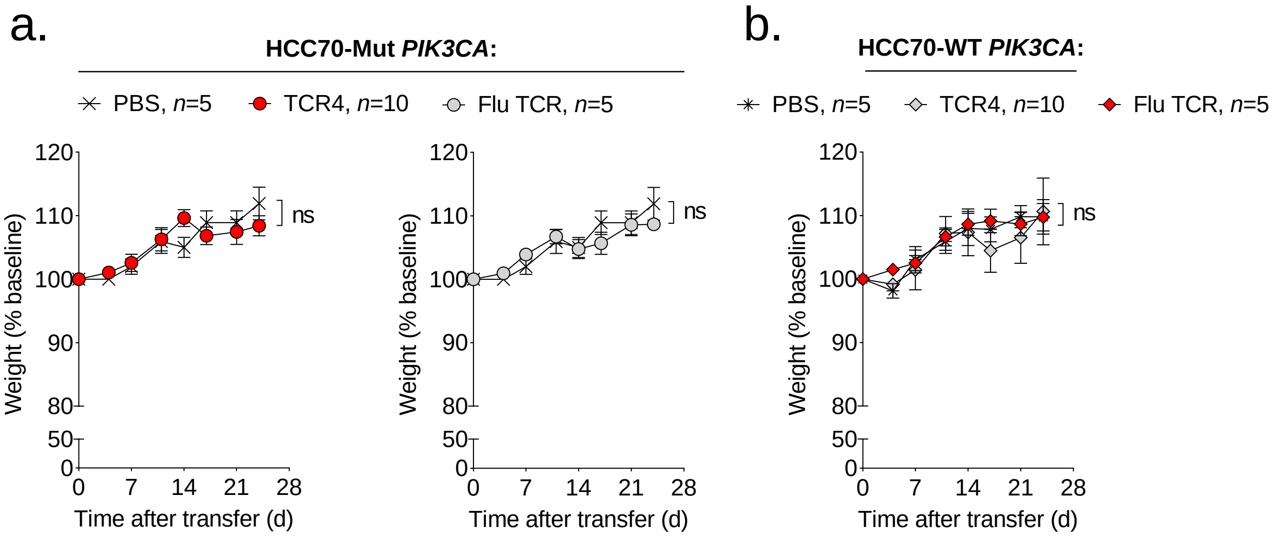

Mice were randomized between indicated treatment groups once subcutaneously implanted HCC70-Mut *PIK3CA* or HCC70-WT *PIK3CA* tumors were established to ~75mm^3. All mice received a twice weekly intraperitoneal injection of 1μg of IL-15 precomplexed to IL-15Rα-Fc (1:1M) and intravenous injection of either phosphate-buffered saline (PBS), CD8$^+$ T cells transduced with an influenza (Flu)-specific TCR, or CD8$^+$ T cells transduced with TCR4. Mice received 7.5e^6 and 2.5e^6 TCR$^+$ T cells on D0 and D3 after randomization. Changes in the weights of mice bearing (**a**) HCC70-Mut *PIK3CA* or (**b**) HCC70-WT *PIK3CA* tumors following treatment. Weights are normalized to the day of randomization. Data shown as mean ± SEM and is representative of two independently performed experiments; TCR4 *n*=10, Flu TCR *n*=5, PBS *n*=5. ns = not significant using a two-way ANOVA test.

Supplementary Fig. 12: Application of the MSK-IMPACT clinical next-gen sequencing (NGS) platform for the efficient identification of *PIK3CA* public NeoAg-expressing cancer patients.

a. b.

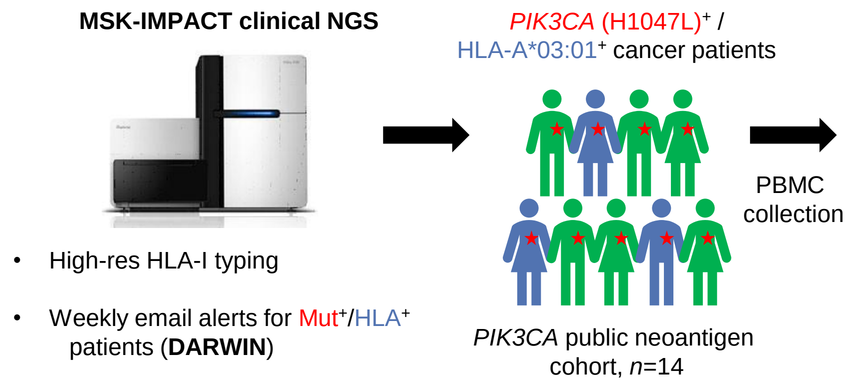

b.

HLA-A	*PIK3CA*	Disease	(*n*)
*03:01	H1047L	BRCA	8
	H1047L	UCEC	1
	H1047L	COAD/ READ	2
	H1047L	Other	3

(**a**) MSK-IMPACT is a CLIA-certified clinical NGS platform capable of performing high resolution HLA-I calls. Using DARWIN, an automated genotype enrollment tool, patients who have a *PIK3CA* (H1047L) (Mut$^+$) cancer and are HLA-A*03:01 (HLA$^+$) were flagged for biospecimen collection using an IRB approved protocol. (**b**) Using this approach, a biotrust of viable peripheral blood mononuclear cells (PBMCs) from a cohort of *n*=14 cancer patients with diverse malignancies who express an identical *PIK3CA* public neoantigen (NeoAg) was collected. (**c**) Summary table of *PIK3CA* public NeoAg expressing cancer types analyzed in this cohort.

Supplementary Fig. 13: Representative gating strategy to determine NeoAg-specific cytokine production by TCR-transduced T cells.

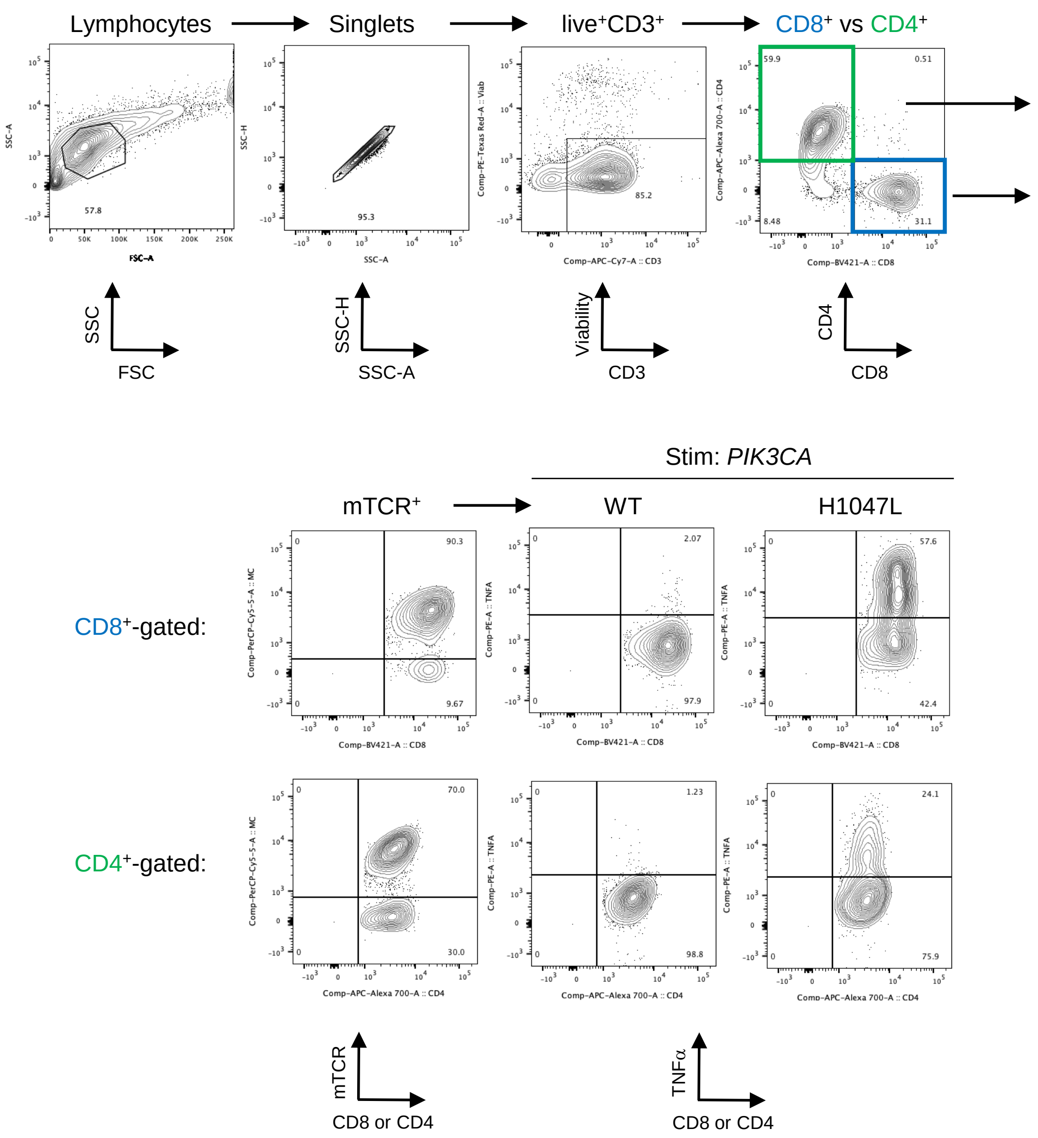

Gating hierarchies and staining results for cytokine production by polyclonal CD4⁺ and CD8⁺ T cells transduced with a murinized T cell receptor following stimulation with wildtype or mutant *PIK3CA*.

Supplementary Fig. 14: Representative gating strategy to assess *PIK3CA* public NeoAg-specific T cells in patient peripheral blood following CD8-enrichment.

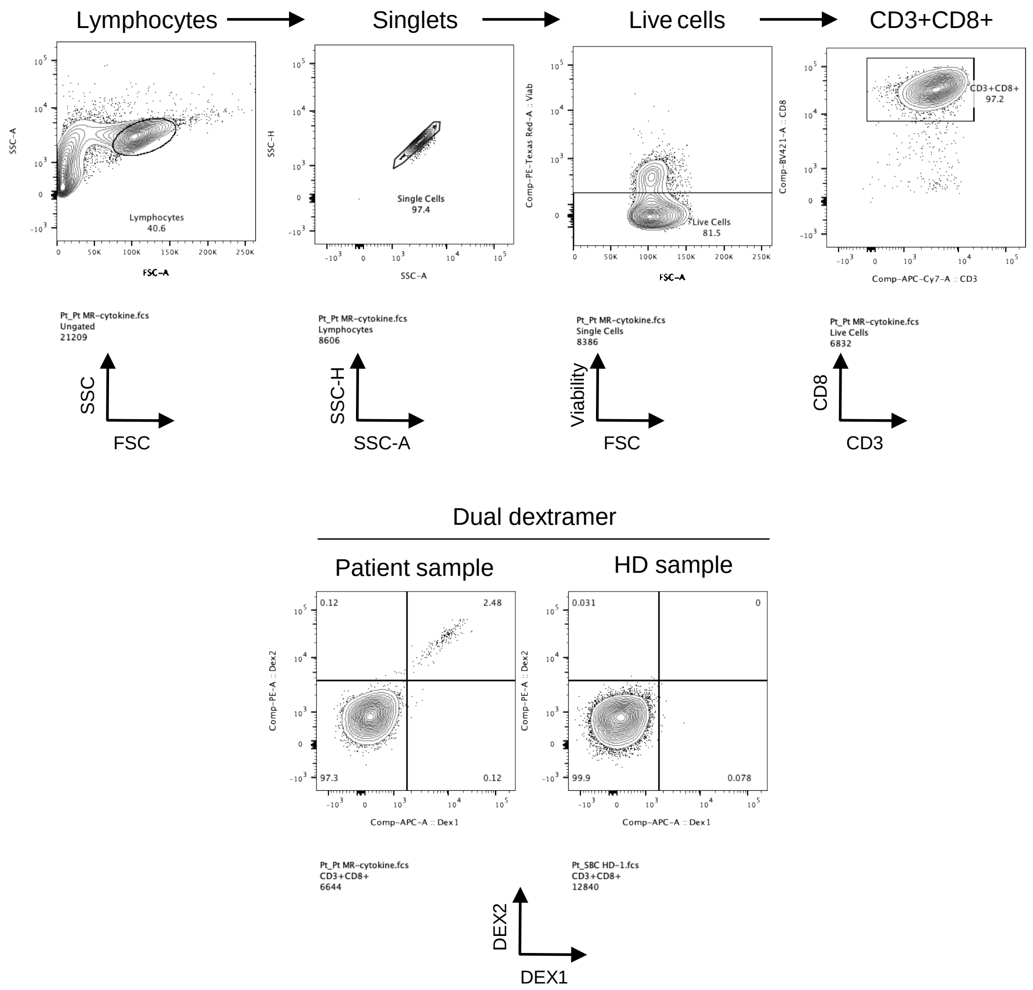

Gating hierarchies and staining results for dual-color HLA-A*03:01 dextramers loaded with the ALHGGWTTK neopeptide on peripheral blood-derived $CD8^+$ T cells.

Supplementary Fig. 15: Representative gating strategy to determine HLA expression on an explanted patient-derived xenograft.

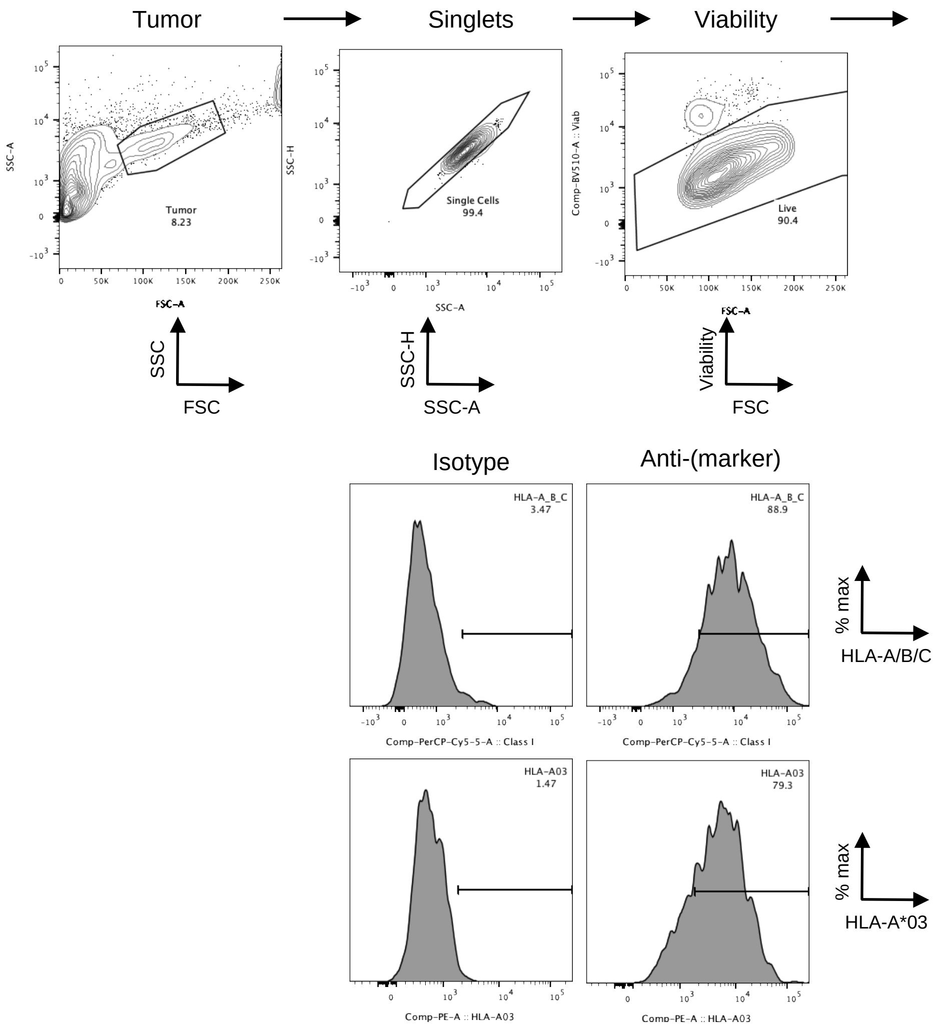

Gating hierarchies and staining results for HLA-I and HLA-A*03 on a single-cell suspension derived from explanted PDX USC_X10.

Supplementary Table 1: High-resolution HLA-A and HLA-DRB1 haplotyping of SIFT-seq screened healthy donors (HDs).

Subject ID	HLA-A, allele 1	HLA-A, allele 2	HLA-DRB1, allele 1	HLA-DRB1, allele 2
HD1	*03:01:01	*69:01:01	*01:01:01	*13:02:01
HD2	*01:01:01	*03:01:01	*11:04:01	*15:01:01
HD3	*03:01:01	*29:02:01	*07:01:01	*07:01:01
HD4	*01:01:01	*03:01:01	*07:01:01	*15:01:01

Supplementary Table 2: X-ray data collection and refinement statistics for the pWT and pMut / HLA-A*03:01 (A*03:01) complexes and the complexes of pMut / A*03:01 with TCR3 or TCR4.

	**pWT/A*03:01**	**pMut/A*03:01**	**TCR3-pMut/A*03:01**	**TCR4-pMut/A*03:01**
**Data Collection**				
Space group	P 6 2 2	P 6 2 2	C 1 2 1	P 43 21 2
Unit cell dimensions				
a, b, c (Å)	156.59, 156.59, 86.21	155.72, 155.72, 85.66	227.27, 46.25, 120.26	72.28, 72.28, 475.73
α, β, γ (°)	90, 90, 120	90, 90, 120	90, 118.67, 90	90, 90, 90
Resolution (Å)	50.00-2.05 (2.09-2.05)	50.00-1.96 (1.99-1.96)	50.00-2.12 (2.16-2.12)	50.00-3.11 (3.15-3.11)
R_{merge}	0.110 (0.598)	0.123 (0.918)	0.111 (0.614)	0.145 (1.365)
$I/\sigma I$	33.7 (2.5)	29.3 (2.6)	17.9 (2.1)	25.0 (2.0)
Completeness (%)	98.9 (89.0)	100 (100)	98.4 (95.8)	99.9 (97.6)
Total reflections	1,579,824	1,911,199	2,333,791	1,856,431
Unique Reflections	39,401 (1745)	44,346 (2187)	61,648 (2969)	24,158 (914)
Redundancy	17.5 (11.7)	17.6 (10.9)	6.2 (5.0)	20.2 (14.1)
**Refinement**				
Resolution (Å)	45.20-2.04	44.95-1.96	49.85-2.12	49.51-3.11
R_{work}/R_{free}	0.195/0.221	0.169/0.205	0.193/0.221	0.198/0.250
No. atoms				
Protein	3430	3484	6913	6512
Average B-factors (Å)	45.0	37.0	54.0	98.0
r.m.s. deviations				
Bond length (Å)	0.002	0.015	0.003	0.002
Bond angles (°)	0.457	1.189	0.593	0.494
Ramachandran favored (%)	98.14	98.94	96.53	97.64
Ramachandran outliers (%)	0	0	0	0
PDB accession code	7L1B	7L1C	7RRG	7L1D

Supplementary Table 3: Assessment of the cross-reactivity potential for TCR4.

Gene	Taxonomy ID	Length	Position	Sequence
PIK3CA (H1047L)	n.a.	1068	1046-1054	A**L**HGGWTTK
WT *PIK3CA*	P42336	1068	1046-1054	A**H**HGGWTTK
TM87B	Q96K49	555	407-415	IVFMGWTTK

Derived from ScanProsite using the motif ‘x-x-x-x-G-W-T-T-K’
n.a. = not applicable

Supplementary Table 4: Overall efficiency of the SIFT-seq platform for retrieving Mut *PIK3CA* public neoantigen-specific TCRs.

SIFT-seq run #	Total number of T cells screened	HD Vs. Patient-derived	Functional TCR retrieved
1	1×10^7	HD	No
2	2×10^7	HD	Yes
3	2.2×10^7	HD	Yes
4	4.5×10^5	HD	No
5	4.5×10^5	HD	Yes
6	2.8×10^6	HD	Yes
7	1.6×10^6	Patient	Yes
	Efficiency of TCR retrieval		5/7 = 71.4%
	# functional TCRs retrieved / T cells screened		1 / $1.1e^7$

Supplementary Table 5: Comparison of the SIFT-seq TCR discovery platform to other neoantigen and TCR discovery approaches.

Reference	Predicted Vs. empiric	Minimal peptide Vs. endogenous processing?	HLA-I Vs. HLA-II?	Screen for WT cross-reactivity?	Healthy donor Vs. patient-derived	Time to TCR retrieval
Wolfl et al., *Nat. Protoc*., 2014	Predicted	Minimal peptide	HLA-I	No	Healthy donor	10-14 days
Gee *et al*., *Cell*, 2018	Empiric	Minimal peptide	HLA-I	Yes	Patient-derived	N.A.
Ali et al., *Nat. Protoc*., 2019	Predicted	Endogenous	HLA-I	No	Healthy donor	2-6 weeks
Peng et al., *Cell Rep*., 2019	Predicted	Minimal peptide	HLA-I	No	Patient-derived	2 days – 4 weeks
Arnaud et al., *Nat Biotechnol*., 2021	Empiric	Endogenous	Both	No	Patient-derived	N.D.
SIFT-seq	Empiric	Endogenous	Both	Yes	Healthy donor and patient-derived	14 days

N.A. = not applicable
N.D. = not described
