# Supplementary material for: Immunogenicity and therapeutic targeting of a public neoantigen derived from mutated PIK3CA
Source: Nat Med. 2022 Apr 28;28(5):946–57. doi: 10.1038/s41591-022-01786-3 (PMC9117146; doi:10.1038/s41591-022-01786-3)
Supplement: Supplementary file 2 — Reporting Summary [file 41591_2022_1786_MOESM2_ESM.pdf]

nature portfolio

Corresponding author(s): Christopher A. Klebanoff
Smita S. Chandran

Last updated by author(s): Mar 12, 2022

# Reporting Summary

Nature Portfolio wishes to improve the reproducibility of the work that we publish. This form provides structure for consistency and transparency in reporting. For further information on Nature Portfolio policies, see our Editorial Policies and the Editorial Policy Checklist.

## Statistics

For all statistical analyses, confirm that the following items are present in the figure legend, table legend, main text, or Methods section.

| n/a | Confirmed | |
|---|---|---|
| ☐ | ☒ | The exact sample size ($n$) for each experimental group/condition, given as a discrete number and unit of measurement |
| ☐ | ☒ | A statement on whether measurements were taken from distinct samples or whether the same sample was measured repeatedly |
| ☐ | ☒ | The statistical test(s) used AND whether they are one- or two-sided<br>*Only common tests should be described solely by name; describe more complex techniques in the Methods section.* |
| ☐ | ☒ | A description of all covariates tested |
| ☐ | ☒ | A description of any assumptions or corrections, such as tests of normality and adjustment for multiple comparisons |
| ☐ | ☒ | A full description of the statistical parameters including central tendency (e.g. means) or other basic estimates (e.g. regression coefficient) AND variation (e.g. standard deviation) or associated estimates of uncertainty (e.g. confidence intervals) |
| ☐ | ☒ | For null hypothesis testing, the test statistic (e.g. $F$, $t$, $r$) with confidence intervals, effect sizes, degrees of freedom and $P$ value noted<br>*Give P values as exact values whenever suitable.* |
| ☒ | ☐ | For Bayesian analysis, information on the choice of priors and Markov chain Monte Carlo settings |
| ☒ | ☐ | For hierarchical and complex designs, identification of the appropriate level for tests and full reporting of outcomes |
| ☒ | ☐ | Estimates of effect sizes (e.g. Cohen's $d$, Pearson's $r$), indicating how they were calculated |

*Our web collection on statistics for biologists contains articles on many of the points above.*

## Software and code

Policy information about availability of computer code

| | |
|---|---|
| Data collection | Single-cell transcriptomic and V(D)J sequencing data was collected using the 10x Genomics immunoprofiling platform. Flow cytometry data was collected using BD FACSDIVA. Cytolytic impedance curves were collected using the ACEA RTCA Software Pro. UniProtKB/Swiss-Prot database sequences were queried for a predetermined motif using ScanProsite Pro. Diffraction data were processed with HKL2000 and solved by molecular replacement via Phaser in Phenix. SPR data was collected on Biacore T200. |
| Data analysis | FlowJo v10.6.2 was used to analyze flow cytometry data. Skyline v4.2 and Byonic v2.7.84 were used to analyze mass-spectrometry data. p/MHC stability was analyzed using OriginPro and MATLAB. SPR data was analyzed with OriginPro. The peptide and CDR loops were removed from the models before molecular replacement and manually rebuilt in Coot after the model was obtained from PHENIX Autobuild. Models were further refined automatically in PHENIX and manually in Coot. Structures were visualized using PyMOL 2.3.4 and Discovery Studio 2019. CCF calls were made using FACETS. HLA genotypes and LOH were inferred using POLYSOLVER and LOHHLA, respectively. Demultiplexing and alignments of single-cell sequencing data were processed using the 10x Genomics Cell Ranger v2.1.1. V(D)J sequences were analyzed on the 10x Loupe V(D)J Browser v4.0. Custom R scripts were developed to aggregate matched sequencing data sets. Custom codes to analyze SIFT-seq data is available on github at https://github.com/abcwcm/Chandran2021 |

For manuscripts utilizing custom algorithms or software that are central to the research but not yet described in published literature, software must be made available to editors and reviewers. We strongly encourage code deposition in a community repository (e.g. GitHub). See the Nature Portfolio guidelines for submitting code & software for further information.

## Data

Policy information about availability of data

All manuscripts must include a data availability statement. This statement should provide the following information, where applicable:

- Accession codes, unique identifiers, or web links for publicly available datasets
- A description of any restrictions on data availability
- For clinical datasets or third party data, please ensure that the statement adheres to our policy

Single-cell RNA-seq datasets are deposited in the Gene Expression Omnibus (GEO) database (GSE172403). Structural data, including coordinates and structure factors of pWT/HLA-I, pMut/HLA-I and the ternary TCR4/pMut/HLA-I and TCR3/pMut/HLA-I complexes are available at the Protein Data Bank (https://www.rcsb.org/) under PDB accession codes 7L1B, 7L1C, 7L1D, and 7RRG. The search model for the HLA-A*03:01 complexes was PDB 2XPG.

# Field-specific reporting

Please select the one below that is the best fit for your research. If you are not sure, read the appropriate sections before making your selection.

☒ Life sciences ☐ Behavioural & social sciences ☐ Ecological, evolutionary & environmental sciences

For a reference copy of the document with all sections, see nature.com/documents/nr-reporting-summary-flat.pdf

# Life sciences study design

All studies must disclose on these points even when the disclosure is negative.

| | |
|---|---|
| Sample size | In vitro experimental data reported were generated from >/= 2 independent experiments containing n=3 biological replicates per condition. Groups of n=5 or n=10 mice per condition were used for in vivo experiments; two independent experiments were performed. All patients expressing HLA-A*03:01 and PIK3CA(H1047L) were consented under an MSKCC Institutional Review Board (IRB)-approved biospecimen umbrella protocol (protocol 12-245; ClinicalTrials.gov ID: NCT01775072), no patients were excluded. |
| Data exclusions | No data were excluded from analyses. |
| Replication | Experiments were repeated >/= 2 times and contained n=3 replicates per biological condition. For in vivo mouse experiments, treatment groups had n=10 mice and control groups had n=5 mice. Experiments were repeated 2 times. All attempts at replication were successful. |
| Randomization | For in vivo mice experiments, mice were randomized into three groups after tumor implantation. Randomization was performed in a blinded fashion. Primary lymphocytes were obtained from anonymous healthy donors. |
| Blinding | For in vivo mouse experiments, investigators were blinded to group randomization for both data acquisition and data analysis. |

# Reporting for specific materials, systems and methods

We require information from authors about some types of materials, experimental systems and methods used in many studies. Here, indicate whether each material, system or method listed is relevant to your study. If you are not sure if a list item applies to your research, read the appropriate section before selecting a response.

## Materials & experimental systems

| n/a | Involved in the study | |
|---|---|---|
| ☐ | ☒ | Antibodies |
| ☐ | ☒ | Eukaryotic cell lines |
| ☒ | ☐ | Palaeontology and archaeology |
| ☐ | ☒ | Animals and other organisms |
| ☐ | ☒ | Human research participants |
| ☒ | ☐ | Clinical data |
| ☒ | ☐ | Dual use research of concern |

## Methods

| n/a | Involved in the study | |
|---|---|---|
| ☒ | ☐ | ChIP-seq |
| ☐ | ☒ | Flow cytometry |
| ☒ | ☐ | MRI-based neuroimaging |

## Antibodies

| | |
|---|---|
| Antibodies used | anti-CD3-APC-H7 (Clone SK7, BD 641397 ), anti-CD4-Alexa Fluor 700 (Clone RPA-T4, Invitrogen 56-0049-42), anti-CD8-efluor450 (Clone SK1, Invitrogen 48-0087-42), anti-mouse TCR- PerCpCy5.5 (Clone H57-597, Invitrogen 45-5961-82). anti-IL-2-PE-Cy7 (Clone MQ1-17H12, Invitrogen 25-7029-42), anti-TNFα-PE (Clone Mab11, Invitrogen 12-7349-82), anti-IFN-gamma-FITC (BD 554551) anti-HLA-A/B/C antibody (Clone W6/32, Biolegend Cat 311428), anti-HLA-A/B/C-PerCpCy5.5 (Clone W6/32, Biolegend Cat 311420), anti-HLA-A03-PE (Clone GAP.A3, Invitrogen Cat 12-5754-42), anti-class II (Clone IVA12, MSKCC in-house). All antibodies were used at a |

final concentration of 5ug/mL, unless indicated otherwise.

| | |
|---|---|
| Validation | All antibodies were used as per manufacturer's instructions. Validation and vetted applications of individual antibodies can be accessed on vendor websites by searching for the antibody clones listed. |

# Eukaryotic cell lines

Policy information about cell lines

| | |
|---|---|
| Cell line source(s) | The retroviral packaging line 293-GP was purchased from Takara Bio (catalog no. 631458). COS-7 cells, originally purchased from ATCC, were obtained through an MTA from S. A. Rosenberg (NCI, Bethesda, USA). HCC70 was purchased from ATCC (catalog no. CRL-2315). |
| Authentication | Cell lines were not authenticated. |
| Mycoplasma contamination | All cell lines are routinely tested in-house for mycoplasma and were confirmed to be negative at the time of use. |
| Commonly misidentified lines (See ICLAC register) | None |

# Animals and other organisms

Policy information about studies involving animals; ARRIVE guidelines recommended for reporting animal research

| | |
|---|---|
| Laboratory animals | Four-six week old female NSG mice were purchased from Jackson Labs (Catalog number 005557) . |
| Wild animals | No wild animals were used in this study. |
| Field-collected samples | No field-collected samples were used in this study. |
| Ethics oversight | All animal procedures were performed in accordance with an MSKCC Institutional Animal Care and Use Committee (IACUC) approved protocol. |

Note that full information on the approval of the study protocol must also be provided in the manuscript.

# Human research participants

Policy information about studies involving human research participants

| | |
|---|---|
| Population characteristics | Patients were identified based on their HLA-I and tumor mutation status. All patients expressing HLA-A*03:01 and PIK3CA(H1047) were consented to an IRB-approved research protocol. Cancer type, disease state, and treatment history of consented patients have been identified and reported. |
| Recruitment | Candidate patients were identified based on results from the MSK-Integrated Mutation Profiling of Actionable Cancer Targets (MSK-IMPACT) clinical NGS platform. DARWIN, an automated genotype-driven enrollment tool that matches NGS results with upcoming patient clinic visits, was used to identify PIK3CA (H1047L)+/HLA-A*03:01+ patients for biospecimen collection. Patients were consented under an MSKCC Institutional Review Board-approved biospecimen umbrella protocol (protocol 12-245; ClinicalTrials.gov ID: NCT01775072). All patients expressing HLA-A*03:01 and a PIK3CA(H1047L) tumor were selected, regardless of age, gender, specific cancer diagnose, or treatment history. |
| Ethics oversight | Protocols were reviewed and approved by the MSKCC IRB. |

Note that full information on the approval of the study protocol must also be provided in the manuscript.

# Flow Cytometry

## Plots

Confirm that:

☒ The axis labels state the marker and fluorochrome used (e.g. CD4-FITC).

☒ The axis scales are clearly visible. Include numbers along axes only for bottom left plot of group (a 'group' is an analysis of identical markers).

☒ All plots are contour plots with outliers or pseudocolor plots.

☒ A numerical value for number of cells or percentage (with statistics) is provided.

## Methodology

| | |
|---|---|
| Sample preparation | T cell cocultures were stimulated in the presence of anti-CD107A- BV650 and Golgi Block. PMA-ionomycin stimulation is included as a positive control. Following 6h, cells were washed in 1x PBS and surface labeled with Live/Dead fixable dye, anti-CD3-APC-H7, anti-CD4-Alexa Fluor 700, anti-CD8-efluor450 and anti-mouse TCR- PerCpCy5.5 for 30 min at 4C. Cells were washed with 1x PBS and then fixed and permeabilized for 15’ at 4C. Surface-labeled cells were then washed with 1x perm- |

wash buffer and labeled with anti-IL-2-PE-Cy7, anti-TNFα-PE and anti-IFN-gamma-FITC for 30 minutes at 4°C in perm-wash buffer. Finally, cells were washed with perm-wash buffer and suspended in 2% FBS in PBS prior to acquisition. Single-cell digests from tumor cells were obtained by enzymatic disruption followed by straining through a 100 micron filter. Cells were labeled with Fixable Live/Dead dye, anti HLA-A03-PE and anti-HLA-A/B/C-PerCpCy5.5 for 30' and 4C, washed and resuspended in 2% FBS in PBS prior to acquisition.

Instrument

BD X20 LSR Fortessa flow cytometer.

Software

Data was collected using BD FACSDIVA and analyzed using FlowJo software version 10.6.2.

Cell population abundance

Enriched CD8 and CD4 populations were obtained using negative selection. Cell purity fractions were determined by flow cytometry to be >90% prior to each experiment.

Gating strategy

Lymphocytes were gated using FSC vs SSC. Downstream gating was determined using a combination of lineage marker and transduced TCR expression to identify sub-populations of interest. Functional gates were assigned based on biological negative controls. Tumor cells were identified based on their FSC and SSC profiles. Gates were assigned based on isotype-labeled controls on live singlet cells.

☒ Tick this box to confirm that a figure exemplifying the gating strategy is provided in the Supplementary Information.
